# Iron Age: Ionic-Liquid-Mediated Interfacial Charge Transfer Enables Selective CO_2_ Photoreduction to Formic Acid on Iron Oxide

**DOI:** 10.1021/jacs.6c04801

**Published:** 2026-05-17

**Authors:** Muhammad I. Qadir, Blendo A. da Silva, Sherdil Khan, Renato B. Pontes, Fabiano S. Rodembusch, Fabiano Mesquita, Brenno A. D. Neto, Paulo E. N. de Souza, Jairton Dupont

**Affiliations:** † 28124 Institute of Chemistry-Universidade Federal Do Rio Grande Do Sul-UFRGS-Av. Bento Gonçalves, 9500 Porto Alegre, 91501-970 Porto Alegre, RS, Brazil; ‡ 67824 Instituto de Química-Universidade Federal de Goiás-UFG-Av. Esperança s/n, Câmpus Samambaia. 74690-900 Goiânia, Goiás, Brazil; § 133642 Institute of Physics, Universidade Federal Do Rio Grande Do Sul (UFRGS), Av. Bento Gonçalves, 9500, Porto Alegre 91501-970 RS, Brazil; ∥ Instituto de Fisica-Universidade Federal de Goiás-UFG-Av. Esperança s/n, Campus Samambaia, 74690-900 Goiânia, Goiás, Brazil; ⊥ Laboratory of Medicinal and Technological Chemistry, University of Brasília, Chemistry Institute (IQ-UnB), Campus Universitário Darcy Ribeiro, Brasília, Distrito Federal 70910-900, Brazil; # Universidade Estadual de Goiás, Molecular Sciences Graduate Program, Anápolis, GO 75132-400, Brazil; ¶ Departamento de Bioquímica y Biología Molecular B e Inmunología Facultad de Química, Universidad de Murcia, P.O. Box 4021, E-30100 Murcia, Spain

## Abstract

Artificial photosynthesis enables the solar-driven reduction of CO_2_ in water to form formic acid, a C_1_ hydrogen carrier and renewable fuel precursor. However, selective formic acid formation in aqueous media remains fundamentally limited by inefficient interfacial proton–electron coupling. Here, we show that ionic liquids (ILs) actively mediate interfacial charge transfer through ion-pair pathways that extract and stabilize photogenerated electrons and protons, thereby enabling selective CO_2_ photoreduction. In this context, our iron oxide microrods exhibit high activity for formic acid production in IL-aqueous solutions under LED irradiation. Among the different ILs, 1,2-dimethyl-1-*n*-butyl-imidazolium 2-methylimidazolate (BMMIm.MeIm) affords the highest efficiency, achieving a yield of about 55.4 μmol (554 μmol.g^–1^) of formic acid with >99% selectivity and an apparent quantum yield of 4.4%. Spectroscopic analyses (EPR, NMR, and ex situ FTIR) reveal the formation of [CO_2_]•^–^ and imidazolium-cation radical species, confirming the direct participation of IL in charge extraction and CO_2_ activation. Mössbauer spectroscopy confirmed hematite as the predominant phase and revealed an IL-induced formation of 6–9% reduced iron (Fe(0)/Fe­(I)), indicating partial Fe_2_O_3_ reduction within the microrods. The IL creates an organized interfacial microenvironment that tunes band energetics, promotes charge separation, and stabilizes CO_2_-derived intermediates, while light-induced radical signatures indicate transient interfacial charge-transfer processes that favor selective formate production. This catalytic system also demonstrates efficiency under natural sunlight, producing 27.7 μmol (277 μmol.g^–1^), highlighting its adaptability and robustness. DFT calculations further reveal that IL cation–anion orientation at Fe_2_O_3_ surfaces modulates band energetics and promotes interfacial charge transfer.

## Graphical abstract

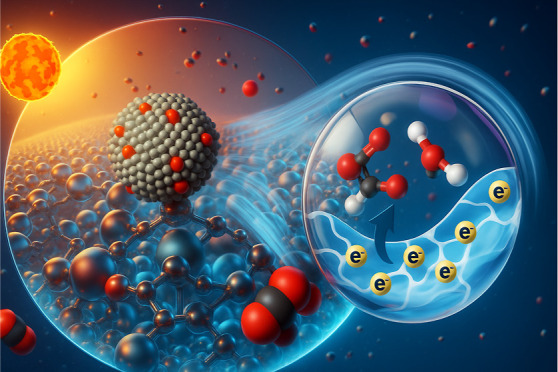

## 1 Introduction

It is widely recognized that one of the most pressing challenges in mitigating global warming is the integration of CO_2_ into a circular economy by incorporating this greenhouse gas into the chemical industry value chain.^1^ A particularly attractive route involves its catalytic reduction to key chemical commodities such as methanol, formic acid, and carbon monoxide.^2^ Among the strategies explored, the direct conversion of solar energy into chemical energy via photocatalytic CO_2_ reduction is especially promising.^3,4^ Different families of catalysts have been investigated: molecular catalysts,^5^ which offer high tunability and valuable mechanistic insights; heterogeneous catalysts,^6^ which provide robustness and scalability; enzymatic and bioinspired catalysts,^7^ which demonstrate remarkable selectivity under mild conditions; and hybrid photoelectrochemical systems,^8^ which most closely approach practical artificial photosynthesis. Ideally, this process would use water as the electron donor, allowing photocatalysts to operate efficiently in aqueous media.^9^

Nevertheless, despite significant advances, only a limited number of photocatalytic systems have been reported that are capable of reducing CO_2_ in water, and even in these cases, the presence of a sacrificial organic reductant is typically still required.^10^ For example, selective CO_2_ photoreduction to formic acid is an attractive strategy for hydrogen storage. To date, sophisticated Ru, Ir, Fe, and Cu MOFs^11–17^ and ruthenium-based molecular catalysts^18–23^ have been reported, typically operating in basic media and as electron–hole scavengers. These systems also require a redox photosensitizer and high-power light sources (300–500 W). However, a significant amount of syngas (5–20%) is also cogenerated along with formates/formic acid. Although ruthenium complexes are active, there is a strong possibility that these molecular systems undergo decomposition under prolonged irradiation. Biohybrid photocatalyst-enzyme systems for NADH regeneration and photocatalytic CO_2_ reduction to formic acid have been reported.^24–28^ However, these systems require photosensitizers and suffer from poor electron-transfer kinetics, which remains a major challenge.

It is worth noting that TiO_2_-based photocatalysts are attractive due to their high photo- and thermal stability as well as their low cost and wide availability. However, their efficiency in CO_2_ photoreduction remains unsatisfactory, largely because of the weak adsorption of CO_2_ on its catalyst surface. In addition, most TiO_2_ photocatalysts are active only under ultraviolet (UV) irradiation. Recently, we demonstrated that a simple impregnation of commercially available TiO_2_ with ILs can modulate the band gap and its position, enhance CO_2_ interaction with the catalyst surface, and significantly improve the photocatalytic performance for both water splitting^29^ and CO_2_ reduction to CO.^30^ We anticipate that the same approach could be used to tune the electronic properties of iron oxides that are used in several catalytic systems. For this purpose, we have selected iron microrods that display broad UV–vis absorption, very fast nonradiative decay, strong photothermal activity, and unique magneto-optical coupling. Their photophysical properties are largely governed by surface oxidation, which introduces semiconducting behavior (Fe_2_O_3_, Fe_3_O_4_) that can support photocatalytic reactions like CO_2_ reduction.^31,32^ We report herein that the simple iron oxide microrods in basic imidazolium ILs allow the reduction of CO_2_ with water to formic acid without the use of any sacrificial agent. Moreover, a dynamic interplay existed between the imidazolium cation and the basic anion of the ILs, which decreased the band gap and facilitated electron extraction, thereby accelerating CO_2_ activation. It is the imidazolium cation that plays a key role in driving CO_2_ activation through the formation of radical reactive intermediates (Scheme 1).

**Scheme 1:**
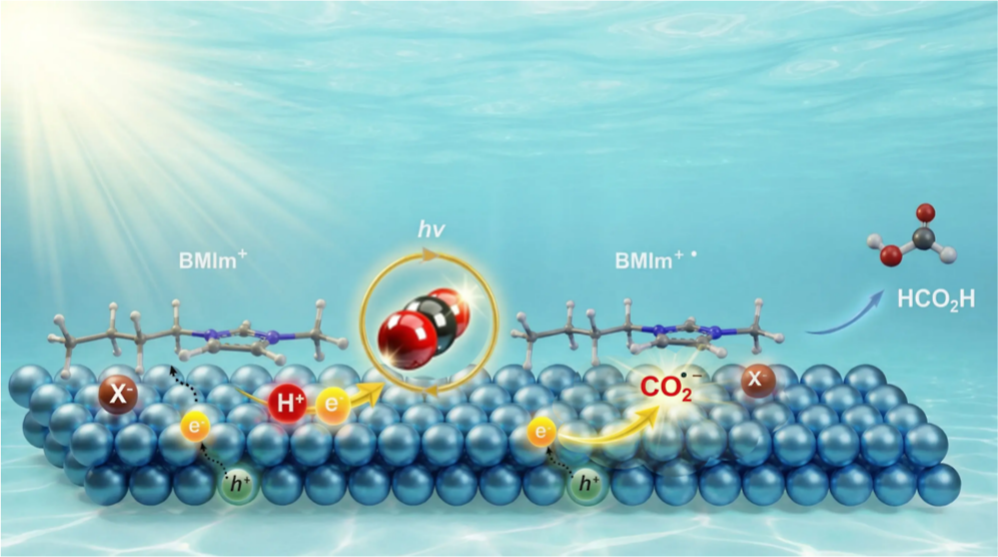
Proposed Ion-Pair-Assisted Mechanism for CO_2_ Photoreduction to Formic Acid at the IL/Fe_2_O_3_ Interface, Illustrating Cooperative Cation–Anion-Mediated Proton-Coupled Electron Transfer and CO_2_ Activation

## 2 Results and Discussion

### 2.1 Catalyst Preparation and Characterizations

Iron oxide (Fe_2_O_3_) microrods were prepared in two steps, as reported earlier.^33^ In the first step, the iron­(II) oxalate complex was prepared by a precipitation method using equimolar amounts of iron­(II) chloride and oxalic acid in *N*,*N*-dimethylacetamide. The obtained yellow precipitate was thermally decomposed, resulting in the formation of Fe_2_O_3_ microrods. The obtained microrods were characterized by SEM and XRD. SEM analysis revealed well-defined rod-shaped structures (Figure 1). PXRD analysis of the as-prepared Fe_2_O_3_ catalyst showed a diffraction pattern characteristic of α-Fe_2_O_3_, with reflections corresponding to the (104), (110), (113), (024), (116), (122), (213), (300), (10 10), and (217) crystalline planes attributed to hematite (Figure S1).

**Figure 1:**
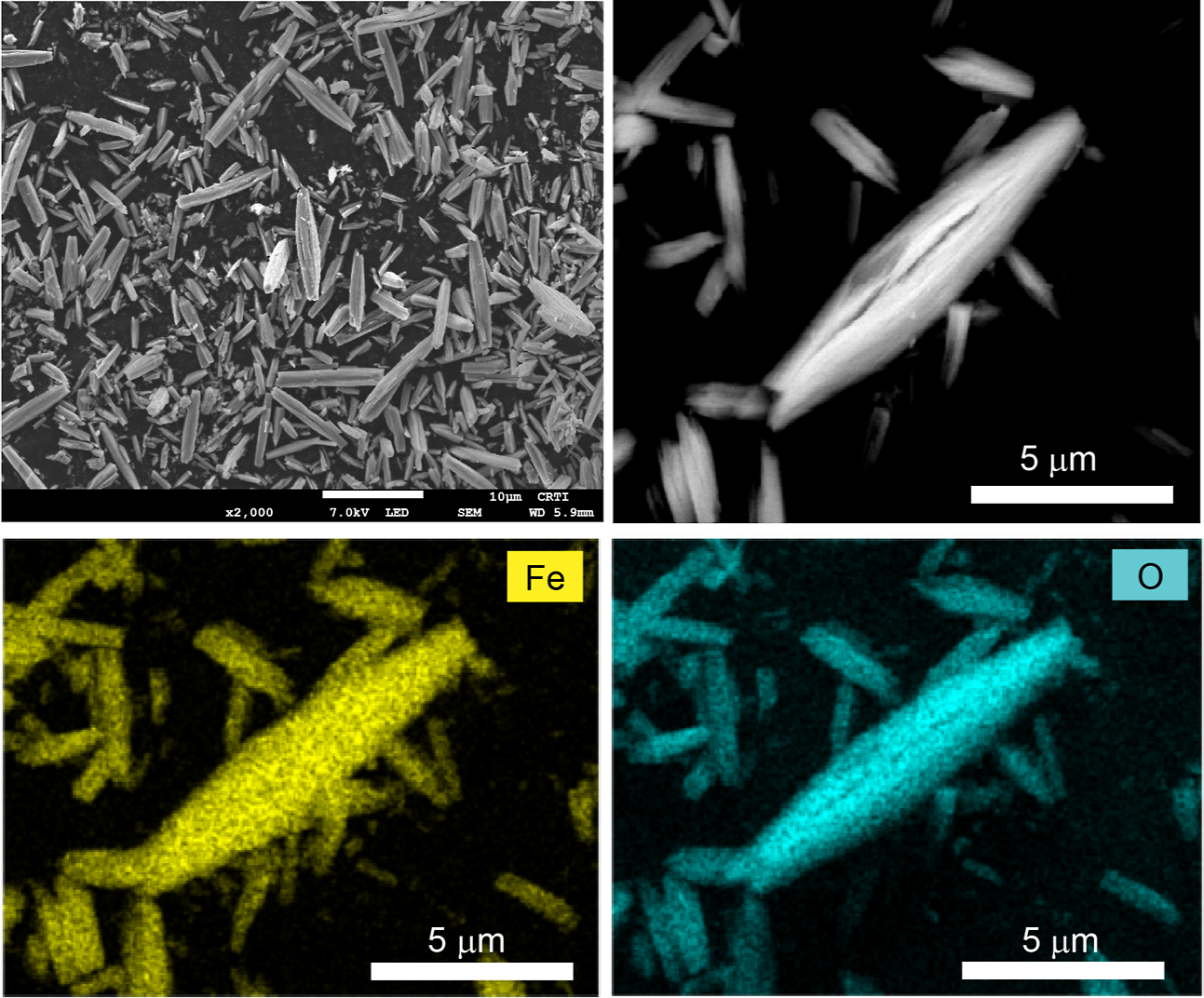
SEM images and elemental mapping of the prepared Fe_2_O_3_ catalyst.

The ILs were impregnated onto pristine Fe_2_O_3_ to investigate the effect of ILs on Fe_2_O_3_. The resulting materials exhibited absorption in the UV–visible region, with maxima around 450 nm, associated with crystal-field transitions and ligand-to-metal charge-transfer mechanisms (Figure 2a).^34,35^ The presence of the IL does not significantly affect the position of the absorption maxima. However, a higher absorption intensity was observed for the sample without IL (pristine Fe_2_O_3_). The presence of the IL led to a decreased intensity in the blue region with the lowest values observed for sample Fe@BMIm.BF_4_.

**Figure 2:**
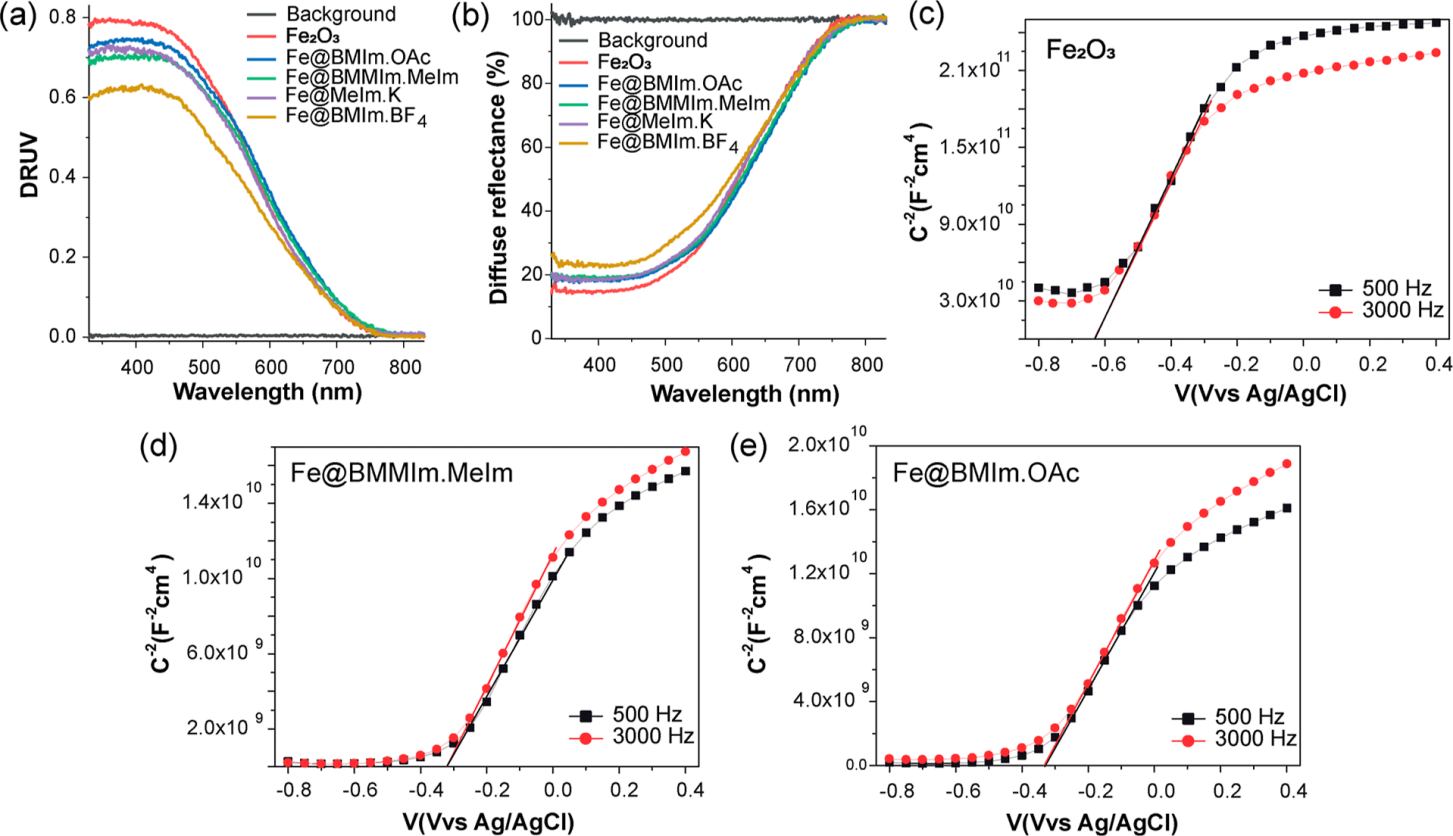
DRUV (a) and diffuse reflectance (b) spectra (330–830 nm) of the iron oxide samples. The respective backgrounds were presented for comparison. (c–e) Mott–Schottky plots of the catalysts.

The IL exhibits absorption in the UV region (∼250 nm), but its contribution to the overall spectra of the Fe_2_O_3_ samples is negligible in the visible range and was therefore not considered in the analysis (Figure 2b). Pristine Fe_2_O_3_ shows an absorption onset at 708 nm (1.75 eV), while IL incorporation induces a slight red shift to 719–732 nm (1.69–1.72 eV), with the most pronounced shifts observed for Fe@BMIm.OAc and Fe@BMIm.BF_4_. These changes suggest modifications in surface states and interfacial electronic interactions rather than significant alterations of the bulk electronic structure.

Consistently, Tauc analysis reveals only minor variations in the apparent bandgap (2.18–2.23 eV) across all IL-modified samples. The largest decrease was observed for Fe@BMIm.OAc (2.18 eV), and Fe@BMIm.BF_4_ remains nearly unchanged relative to that of pristine Fe_2_O_3_, indicating minimal perturbation. The differences between onset- and Tauc-derived values arise from their sensitivity to distinct features, with absorption onset reflecting surface and tail states and Tauc analysis probing bulk electronic transitions.^36,37^ The apparent bandgap values derived from Tauc analysis show only minor variation upon IL incorporation, indicating that the bulk electronic structure of Fe_2_O_3_ remains largely unaffected (Table 1). In contrast, absorption onset shifts are more sensitive to band-tail states, surface states, and interfacial electronic interactions introduced by the IL. Accordingly, these onset shifts should not be interpreted as intrinsic bandgap modifications. The catalytic enhancement observed upon IL incorporation is therefore attributed primarily to interfacial electronic modulation rather than bulk bandgap narrowing. Unlike what has been reported in the literature, the samples did not exhibit photoluminescence upon excitation in the 240–540 nm range at 20 nm intervals, as would be expected.^38^

**Table 1:** Bandgap Values of Iron Oxide Samples Obtained from Solid-State Absorption and Diffuse Reflectance UV–Vis Spectroscopies

Sample	Bandgap (eV)	
	Tauc Plot^a^	λ_abs_^onset^ ^b^
Fe_2_O_3_	2.24	708 nm (1.75 eV)
Fe@BMIm.OAc	2.18	731 nm (1.69 eV)
Fe@BMMIm.MeIm	2.19	727 nm (1.70 eV)
Fe@MeIm.K	2.21	719 nm (1.72 eV)
Fe@BMIm.BF_4_	2.23	732 nm (1.69 eV)

a Diffuse reflectance spectra.

b DRUV spectra, *E*_g_ = 1239/λ_abs_^onset^

It is worth noting that these observations in band gap changes for iron oxide are generally consistent with those calculated for TiO_2_.^39^ In both systems, imidazolium-based ILs can modulate the semiconductor’s electronic structure through interfacial charge transfer: cations tend to induce a downward shift of the band levels by withdrawing electron density, while coordinating or basic anions can counteract this by donating charge to the surface. The slight band gap narrowing observed for Fe_2_O_3_ coated with acetate- or imidazolate-based ILs suggests enhanced electronic coupling and charge delocalization, similar to the upward energetic shift seen in TiO_2_ with coordinating anions, whereas the nearly unchanged band gap for Fe@BMIm.BF_4_ reflects the weakly interacting nature of the fluorinated anion, leaving the cation effect predominant.

Figure 2c–e shows the Mott–Schottky plots of the films, where all samples exhibit positive slopes, confirming n-type behavior. Pristine Fe_2_O_3_ presents a flat-band potential of approximately −0.62 V vs Ag/AgCl, while Fe@BMIm.OAc and Fe@BMMIm.MeIm shift to around −0.37 V, indicating a positive shift. This shift is attributed to electronic modulation induced by IL coordination, which may alter the local environment of Fe^3+^ and influence oxygen vacancy concentration. Mössbauer spectroscopy (see below) confirms that Fe^3+^ remains dominant with a preserved hematite structure, and only minor reduced superparamagnetic Fe species (<10%) are detected in IL-modified samples (see below). Their limited fraction suggests that the flat-band shift mainly arises from electronic modification of the compound. The MS slopes decrease from ∼4.3 × 10^10^ (Fe_2_O_3_) to ∼ 3.0 × 10^10^ (Fe@BMMIm.MeIm) and ∼2.8 × 10^10^ (Fe@BMIm.OAc), corresponding to donor densities (N_D_) of 4.10 × 10^19^, 5.88 × 10^19^, and 6.29 × 10^19^ cm^–3^, respectively. These results indicate that IL incorporation increases defect density, contributing to the observed flat-band shift. This change suggests the downward movement of the conduction band since Mössbauer analyses suggested the presence of reduced Fe species and the Fe_2_O_3_ phase remained mainly persevered; therefore, we suggest the presence of reduced species increases the electronic density of states, resulting in CB tailing. This positive flat-band shift suggests a modification of band-edge alignment at the semiconductor interface. Notably, this shift does not necessarily imply intrinsic bandgap narrowing but rather reflects interfacial electronic modulation induced by IL coordination.^30^

The chemical environment of the surface atoms in the IL-functionalized iron catalysts was investigated by X-ray photoelectron spectroscopy (XPS). The N 1s XPS spectra of the Fe_2_O_3_ catalysts showed a single peak centered at a binding energy of 402.0 eV, which is attributed to the nitrogen atoms within the imidazolium ring.^40,41^ In MeIm IL, an additional peak appeared at 399.0 eV in the N 1s region, alongside the main peak at 402.0 eV (Figure 3b). This new signal is attributed to the formation of Fe–N bonds, indicating coordination between Fe and the nitrogen atoms of the 2-methylimidazolate anion. A similar Fe–N peak was observed in Fe-MOFs where Fe atoms are directly coordinated to the N atoms of 2-methylimidazole.^42,43^ It is worth noting that this Fe–N peak is absent when the catalyst is decorated with BMIm.OAc IL (Figure 3A). The Fe–N peak was also observed in the used catalyst (Figure 3c).

**Figure 3:**
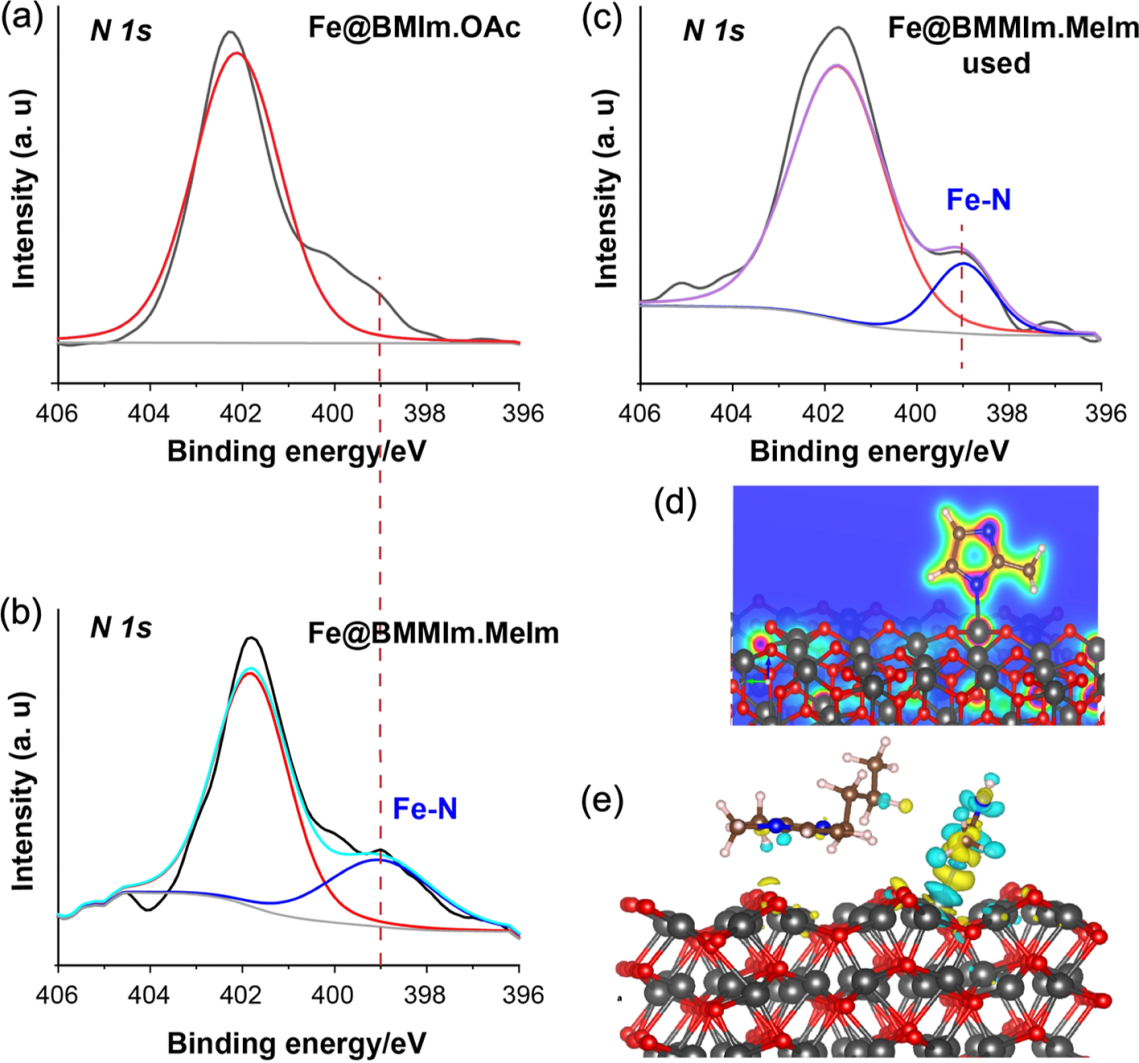
XPS analysis, N 1s region, of (a) Fe@BMIm.OAc, (b) Fe@BMMIm.MeIm, and (c) Fe@BMMIm.MeIm after photocatalysis: black curve (experimental data) and red/blue/cyan curves (main fitted components). (d) Contour plot of the electronic charge density for the imidazole molecule: N atom (blue), O atom (red), and Fe atom (dark gray). (e) Charge density difference for the energetically most stable structure. The yellow and blue represent the increasing and decreasing electron densities, respectively. The isovalue is 0.003 eV/Å^3^.

Figure 3d provides a two-dimensional contour map of the charge density in the adsorption plane, which further evidences orbital hybridization between the nitrogen atoms of the 2-methylimidazolate ring and the Fe sites of the surface. The combined analysis reveals that adsorption between Fe and nitrogen of the 2-methylimidazolate anion is not dominated by weak physisorption but instead involves electronic coupling and partial charge transfer, leading to a stabilized interface (Figure 3d).

The charge density of the BMMIm.MeIm IL onto Fe_2_O_3_ was calculated. Figure 3e depicts the charge density difference isosurface for this lowest-energy adsorption geometry, where yellow and cyan regions correspond to electron accumulation and depletion, respectively, highlighting the redistribution of charge upon binding.

Mössbauer measurements were performed at room temperature, and spectral analysis was carried out by fitting Lorentzian line shapes using the least-squares method (Figure 5). The presence of doublets is related to the stabilization of a superparamagnetic phase of the α-Fe_2_O_3_ compound (hematite) due to relaxation effects arising from particles with reduced sizes, on the order of or less than 10 nm.

**Figure 4:**
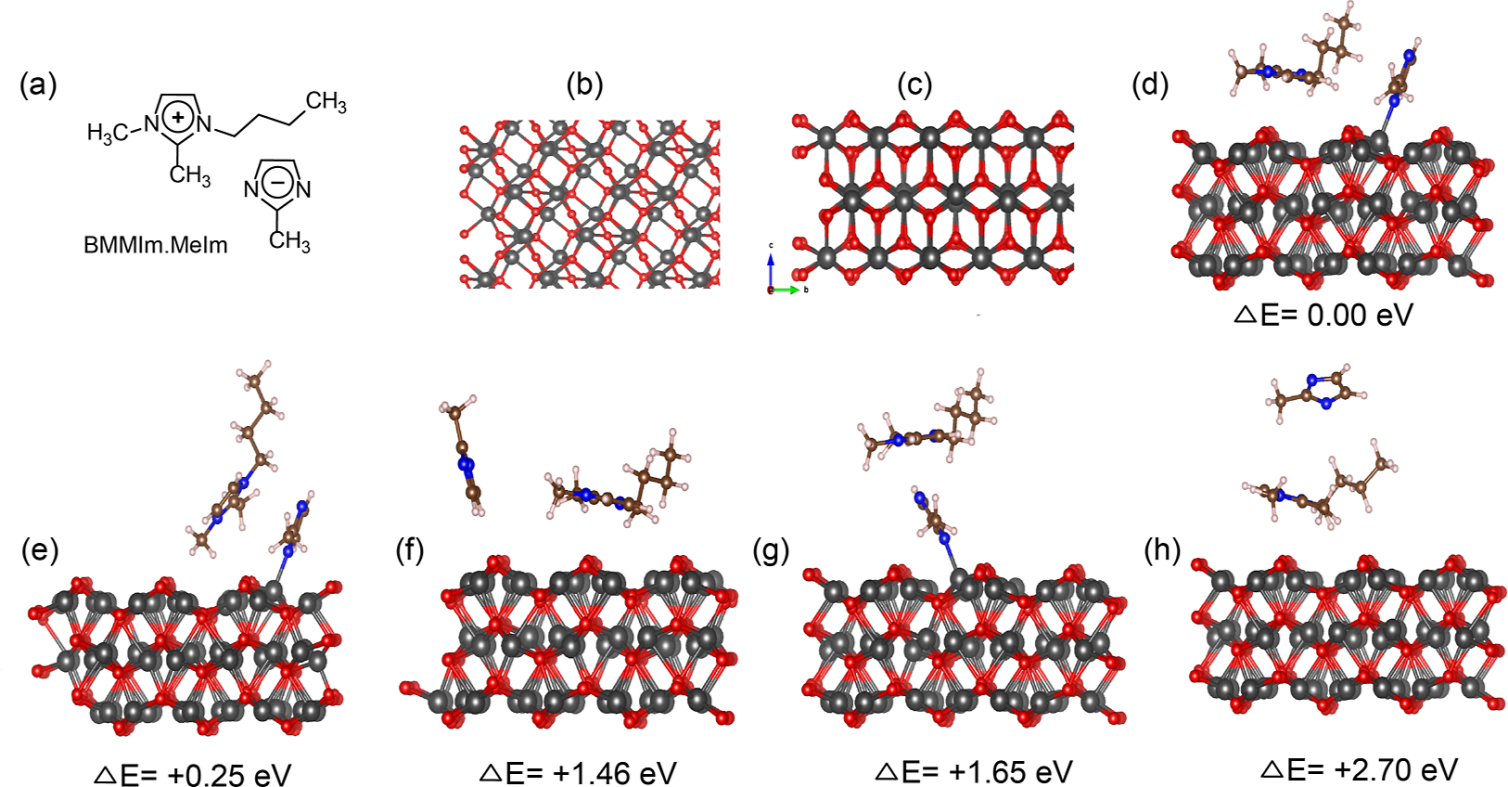
(a) Structure of BMMIm.MeIm IL, (b,c) ball-and-stick representations of the α-Fe_2_O_3_ (110) surface, and (d–h) possible BMMIm.MeIm IL adsorption geometries [relative energies (ΔE)] onto the α-Fe_2_O_3_ (110) surface.

**Figure 5:**
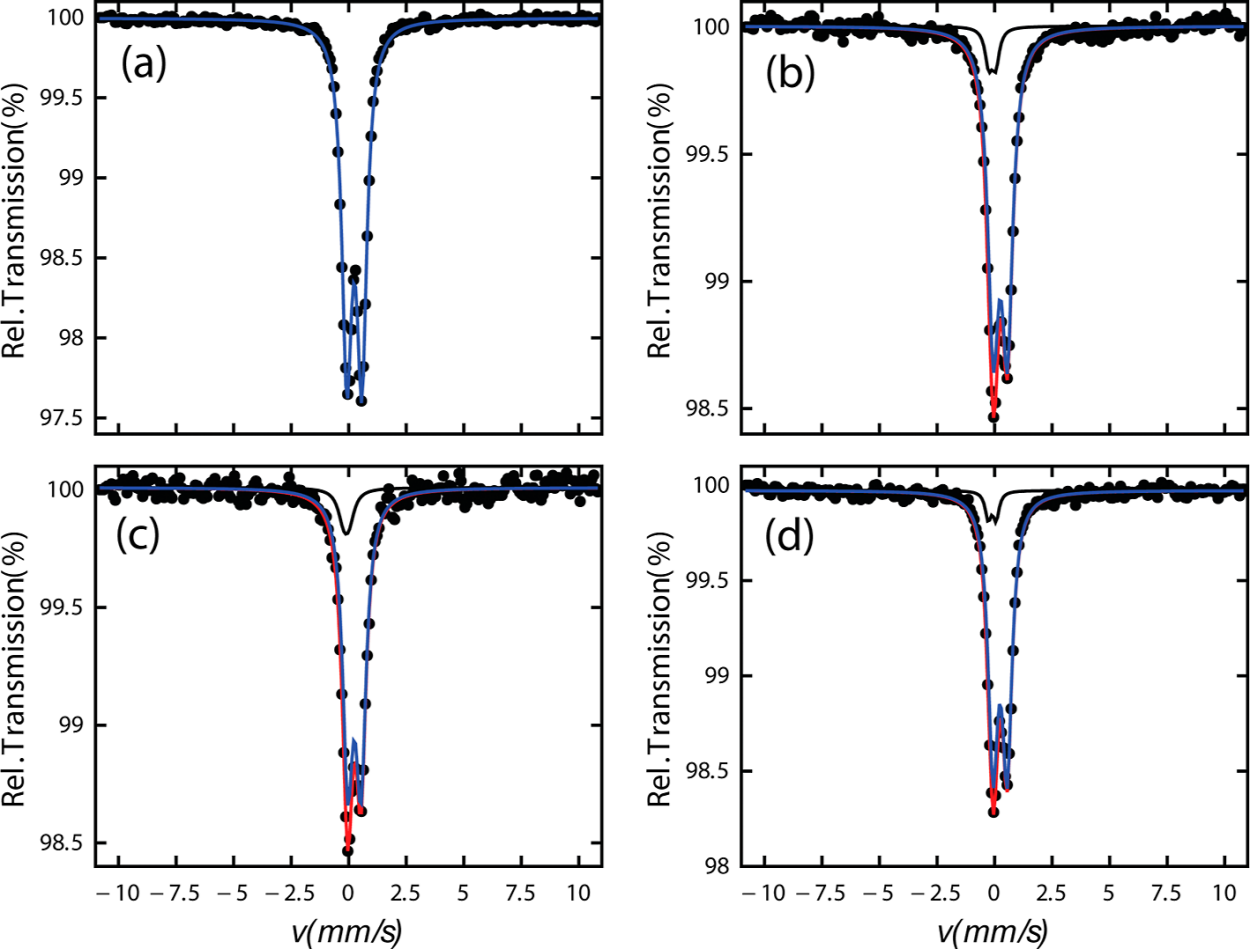
Summary of the results from the analysis of the Mössbauer spectra of (a) Fe_2_O_3_, (b) Fe@BMMIm.MeIm, (c) Fe@BMIm.OAc, and (d) Fe@K.MEIm. The site numbering refers to the hyperfine parameters found in the samples, where Site 1 and Site 2 are related to the superparamagnetic responses of hematite and reduced species (minority phase), respectively.

The spectral areas corresponding to hematite are 100%, 94%, 93%, and 91% for samples pristine Fe_2_O_3_, Fe@K.MeIm, Fe@BMMIm.MeIm, and Fe@BMIm.OAc, respectively. No significant variations were observed in hyperfine parameters for the entire series of studied samples. The values found for (δ) agree with the expected oxidation state for hematite (Fe^3+^), while the higher value of (ΔEQ), compared to the expected, could be related to the reduced particle size.

A second contribution to the Mössbauer spectrum appears in samples Fe@K.MeIm, Fe@BMMIm.MeIm, and Fe@BMIm.OAc (Table S1), where an IS_2_ value of ∼ 0.00 mm/s for all samples indicates the absence of Fe^2+^. However, the increase in the quadrupole splitting value suggests the presence of more reduced iron species, potentially with oxidation states lower than Fe^2+^ (e.g., Fe^+^ and/or Fe^0^).

The physicochemical properties of the catalysts (Table S2) show that Fe_2_O_3_, Fe@BMMIm.MeIm, and Fe@BMIm.OAc possess surface areas of 137.1, 16.8, and 3.1 m^2^ g^–1^, respectively. The decrease in surface area relative to pristine Fe_2_O_3_ is attributed to partial pore filling and surface coverage by the ionic liquids. BJH analysis indicates pore sizes of 4.2, 11.2, and 3.5 nm for Fe_2_O_3_, Fe@BMMIm.MeIm, and Fe@BMIm.OAc, respectively, reflecting modifications in pore accessibility upon IL incorporation. Although the incorporation of ILs significantly reduces the measured BET surface area due to partial pore filling and surface coverage, the catalytic activity does not follow the same trend. This observation indicates that the reaction is governed primarily by the interfacial chemical environment created by the ionic liquid rather than by the geometric surface area alone. The IL layer can promote CO_2_ capture, stabilize charged intermediates, and regulate local proton activity, thereby enhancing the efficiency of the active sites at the Fe_2_O_3_/IL interface. Consequently, the catalytic performance is determined more by the nature and reactivity of these interfacial sites than by the total accessible surface area measured by N_2_ adsorption.

Density functional theory (DFT) calculations were performed to investigate the adsorption geometries of BMMIm.MeIm IL on the Fe_2_O_3_ (110) surface (Figure 4). Figure 4a,b shows the atomic structure of the clean Fe_2_O_3_ (110) slab viewed along different crystallographic directions, highlighting the surface termination. For this surface, the corresponding minimum distances of three differently coordinated oxygen atoms with the nearest metal atom are 1.79 Å, 1.93 Å, and 2.13 Å for two-, three-, and four-folded coordinated oxygen atoms, in good agreement with a previous investigation.^44^ Figure 4d–h illustrates five optimized configurations for the adsorption process, in which the molecules were placed in distinct orientations and positions relative to the hematite surface in order to explore possible binding sites and interaction modes. The increasing positive Δ*E* values indicate less favorable adsorption orientations. Thus, among them, configuration in Figure 4d is clearly identified as the most stable reference state (ΔE = 0.00 eV). A slightly less favorable orientation is found in Figure 4e with Δ*E* = +0.25 eV, while configurations in Figures 4f and 4g are considerably less stable, with relative energies of +1.46 eV and +1.65 eV, respectively. The configuration in Figure 4h, with Δ*E* = +2.70 eV, is the least favorable. Focusing on the most stable structure, the 2-methylimidazolate anion stays almost perpendicular to the surface with a bond distance between N and Fe atoms of ∼1.95 Å. In 1,2-dimethyl-3-butyl-imidazolium, the imidazole ring is parallel to the Fe_2_O_3_ (110) surface, and the shortest calculated distance between an H atom and a surface oxygen is 2.34 Å. Also, the smaller distance between such molecules is ∼2.90 Å (C^···^H distance).

### 2.2 Selective Catalytic CO_2_ Photoreduction

The photoreduction of CO_2_ (1 bar) to HCO_2_H over an Fe_2_O_3_ catalyst was carried out in a 220 mL Fischer–Porter glass reactor using various ILs in a CH_3_CN/H_2_O mixture, without any sacrificial agent. The obtained results under LED of 365 nm and 10 W LED (18 mW.cm^–2^) white-cold irradiations are summarized in Table 2 and Figure 6a. No gaseous CO and H_2_ and HCO_2_H were detected when only Fe_2_O_3_ and BMMIm.MeIm IL were used, indicating that neither the Fe_2_O_3_ catalyst nor the IL is capable of driving the overall reaction independently (Table 2, entries 13–15). Interestingly, in the dark, the Fe_2_O_3_ catalyst in BMMIm.MeIM and BMIm.OAc IL solutions showed the formation of formic acid of about 14.6 and 10.7 μmol, corresponding to specific yield values of 146 and 107, respectively (Table 2, entries 1 and 2).

**Table 2:** Selective Photoreduction of CO_2_ to Formic Acid by Fe_2_O_3_ under LED Irradiations^a^

En	hv	IL	FA (μmol)	CO/H_2_ (μmol)
1	-	BMMIm.MeIm	14.6 ± 4.1	-
2	-	BMIm.OAc	10.7 ± 2.0	-
3	365 nm	BMIm.OAc	14.2 ± 1.3	trace
4		BMMIm.MeIm	21.0 ± 1.9	trace
5		P4442.Im	9.7 ± 3.2	trace
6		K.MeIm	-	-
7		BMIm.BF_4_	-	-
8	10W	BMMIm.MeIm	24.7 ± 4.6	trace
9		BMIm.OAc	16.1 ± 0.5	trace
10		K.MeIm	-	-
11		BMIm.BF_4_	-	-
12^b^		BMMIm.MeIm	55.4 ± 4.0	trace
13		BMMIm.OAc	15.0	traces
14	10W, 365 nm	-	-	-
15		BMMIm.MeIm	-	-
16		BMIm.OAc	-	-

a Reaction conditions: (a) Cat. (100 mg), IL (0.75 mmol), CO_2_ (1.0 bar, 200 mL reactor), CH_3_CN (2.5 mL), H_2_O (0.5 mL), temp. (27 ± 2 °C), time (5 h), and LED (10 W, white).

b Cat. (100 mg), IL (1.92 mmol), CO_2_ (1.0 bar), CH_3_CN (2.5 mL), H_2_O (0.5 mL), temp. (27 ± 2 °C), time (5 h), and LED (10 W, white). FA (amount of formic acid produced using the added IL).

**Figure 6:**
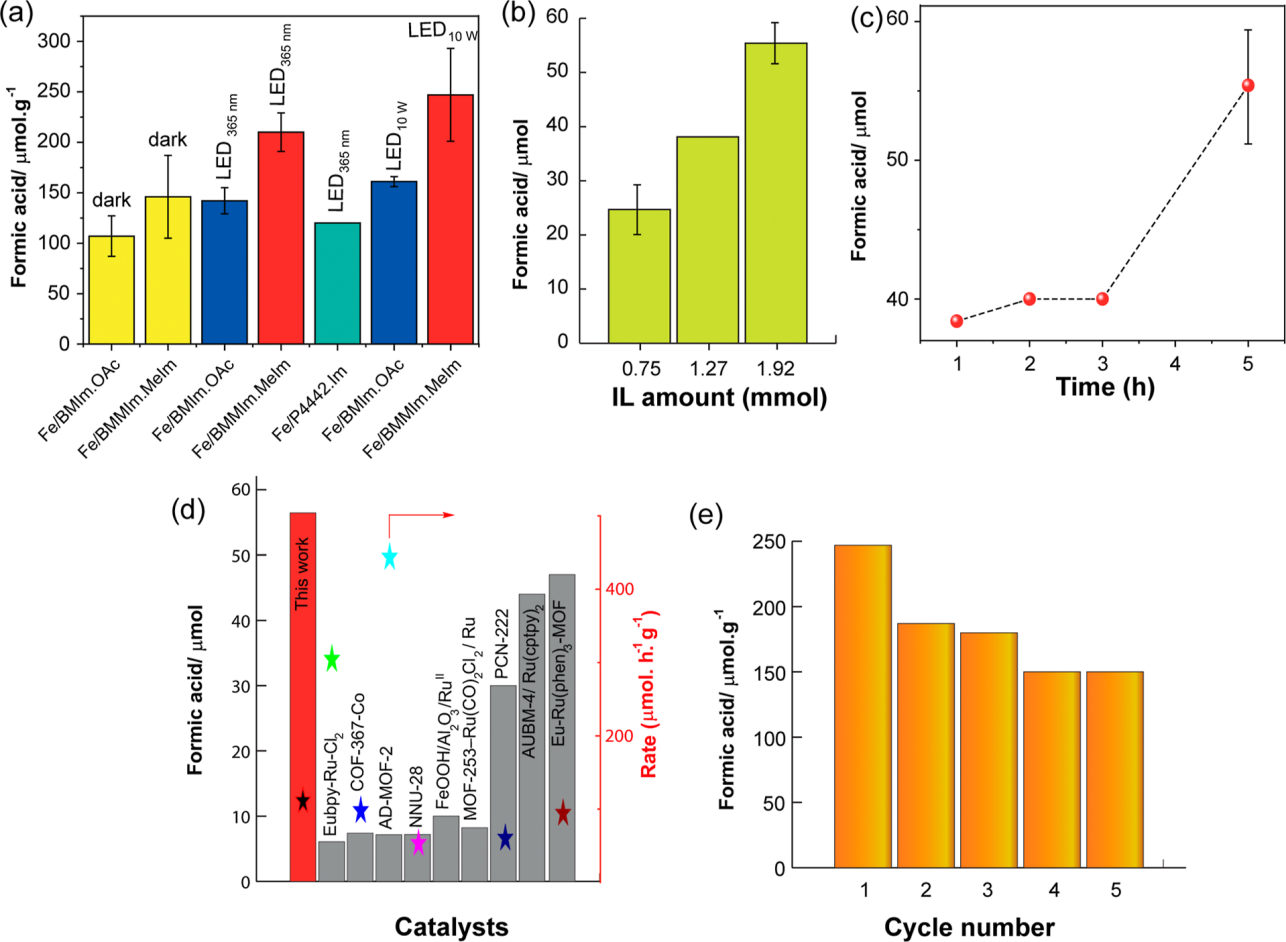
Reaction conditions: (a) specific yield for formic acid in different IL solutions; Cat. (100 mg), IL (0.75 mmol), CO_2_ (1.0 bar, 220 mL reactor), CH_3_CN (2.5 mL), H_2_O (0.5 mL), temp. (27 ± 2 °C), time (5 h), and LED (10 W, white). (b) Formic acid yield vs BMMIm.MeIm IL amount. (c) Formic acid yield vs time in BMMIm.MeIm IL (1.92 mmol) solution. (d) Literature comparison of catalytic performance. Stars represent activity expressed as rate (μmol.h^–1^.g^–1^). Absence of a star indicates that the rate was not reported in the original reference (see Table S2). (e) Recycling in BMMIm.MeIm IL (0.75 mmol) solution.

Under LED irradiation (λ 365 nm), an increase in the yield of formic acid was achieved. The formic acid yield in the BMMIm.MeIm IL solution increased to 44% with 21.0 ± 1.9 μmol, while in the BMIm.OAc IL solution, it augmented to 33% with 14.2 ± 1.3 μmol (Table 2, entries 3 and 4). It is important to note in all cases, >99% selectivity to formic acid was observed. Our Fe_2_O_3_ catalyst in the IL solution of BMIm.BF_4_ IL did not produce HCO_2_H, CO, or H_2_ (Table 2, entry 7).

Of note is the comparative influence of the different cations with the imidazolate anion, such as potassium (potassium 2-methylimidazolate, K.MeIm) and phosphonium tri­(*n*-butyl)-ethylphosphonium imidazolate, P4442.Im), which showed that the Fe_2_O_3_ in the imidazolium cation has superior activity as compared to others. When the imidazolium cation is replaced with a potassium cation, no formic acid generation is observed in the K.MeIm solution (Table 2, entry 6), while 9.7 ± 3.2 μmol of formic acid is obtained with phosphonium-based IL (P4442.Im), as shown in Table 2, entry 5. This is an indication that the strength of the contact ion pair is related to the strength of IL charge transfer, which is more pronounced for BMMIm.MeIm than for the other cations associated with the imidazolate anion. Accordingly, although the P_4442_.Im IL shows measurable activity, it remains less active than imidazolium-based ILs, consistent with the role of imidazolium cations in promoting a favorable interfacial environment for CO_2_ activation. Moreover, BMMIm.MeIm, which forms the most stable contact ion pair,^45^ causes an energetic upward shift of the valence and conduction band edge, simultaneously decreasing the band gap. This energetic shift of the states can be attributed to the amount of charge transferred from the IL to the Fe_2_O_3_ surface.

Of note, an increase in the light intensity leads to the augmentation in yield of formic acid with maximum selectivity (>99%). When the reactions were performed in a 10 W LED white cold lamp (intensity 18 mW.cm^–2^), an increase in the formic acid was observed. Under these conditions, the Fe_2_O_3_ catalyst produced 24.7, 16.1, and 15.0 μmol of formic acid in the BMMIm·MeIm, BMIm·OAc, and BMMIm·OAc ILs, respectively (Table 2, entries 8, 9, and 13), underscoring the role of anion basicity in modulating the system, either by shifting the equilibrium toward formate formation or bicarbonate^46^ or by promoting the generation of NHC species.^47^ Notably, the temperature of the reaction medium increased under irradiation, reaching approximately 42 °C (Figure S5).

Because Fe_2_O_3_ exhibits nonradiative relaxation, particularly in its hematite (α- Fe_2_O_3_),^48^ phase illumination produces measurable heating of the reaction medium (∼42 °C, Figure S5). To differentiate between photochemical and photothermal contributions, control experiments were performed in the dark at temperatures matching those reached during irradiation (42 °C). While increasing the reaction temperature from 25 to 42 °C in the dark resulted in a marginal increase in yield from 14.6 ± 4.1 μmol to 16.1 ± 1.8 μmol, this thermal effect accounts for only a small fraction of the total activity. In contrast, under identical in situ thermal conditions (42 °C) with light irradiation, the yield significantly increased to 21.0 ± 1.9 μmol (Table 2, entry 4), confirming that light-driven charge-carrier generation is essential. This clear divergence between the temperature-matched dark experiment and the illuminated reaction indicates that light-induced charge-carrier generation plays a dominant role in driving the reaction. These results demonstrate that the process proceeds predominantly via a photoassisted pathway, with photothermal effects contributing only a secondary kinetic enhancement.

The ILs have been reported as effective electron acceptors and can significantly accelerate electron transfer kinetics. The effect of the amount of BMMIm.MeIm IL was investigated, showing an increase in formic acid yield with increasing IL concentration (Figure 6b). The formic acid yield reached 55.4 ± 4.0 μmol. Our catalytic system achieved a higher formic acid production with >99% selectivity compared to previously reported heterogeneous Ru-based catalysts (Figure 6d, Table S1).^11,49–57^ The apparent quantum yield (AQY) for formic acid formation was 4.4%. The unique combination of sacrificial-agent-free operation, > 99% selectivity, and efficient performance under natural sunlight positions this system beyond typical benchmark laboratory-scale photocatalytic systems. These features highlight IL-mediated interfacial charge transfer as the governing principle.

To confirm that formic acid originates exclusively from CO_2_ reduction rather than IL degradation or adventitious carbon sources, additional isotopic and integrity controls were performed. ^13^CO_2_-labeling experiments yield the characteristic enriched ^13^C resonance at 168.2 ppm corresponding to H^13^COOH (Figure 7a). Further validation was obtained through the ^1^H NMR spectrum (Figure 7b), which displayed a doublet at 8.25 and 8.45 ppm with equal intensity, the expected ^1^H–^13^C coupling pattern (^1^J_CH_ = 80 Hz, confirming incorporation of carbon from CO_2_. In contrast, a control experiment conducted under identical conditions using unlabeled CO_2_ shows no enhanced signal in the formate region of the ^13^C NMR spectrum (Figure S9), excluding alternative carbon sources. Furthermore, ^1^H and ^13^C NMR spectra of the recovered IL/solvent after irradiation reveal no new resonances or degradation products (Figures S8 and S9), demonstrating its chemical integrity during catalysis. Collectively, these results provide strong evidence that formic acid formation arises solely from CO_2_ reduction rather than IL decomposition or surface contaminants. Of note, no degradation of IL was observed by ^1^H NMR experiments after use (see Figures S8 and S11).

**Figure 7:**
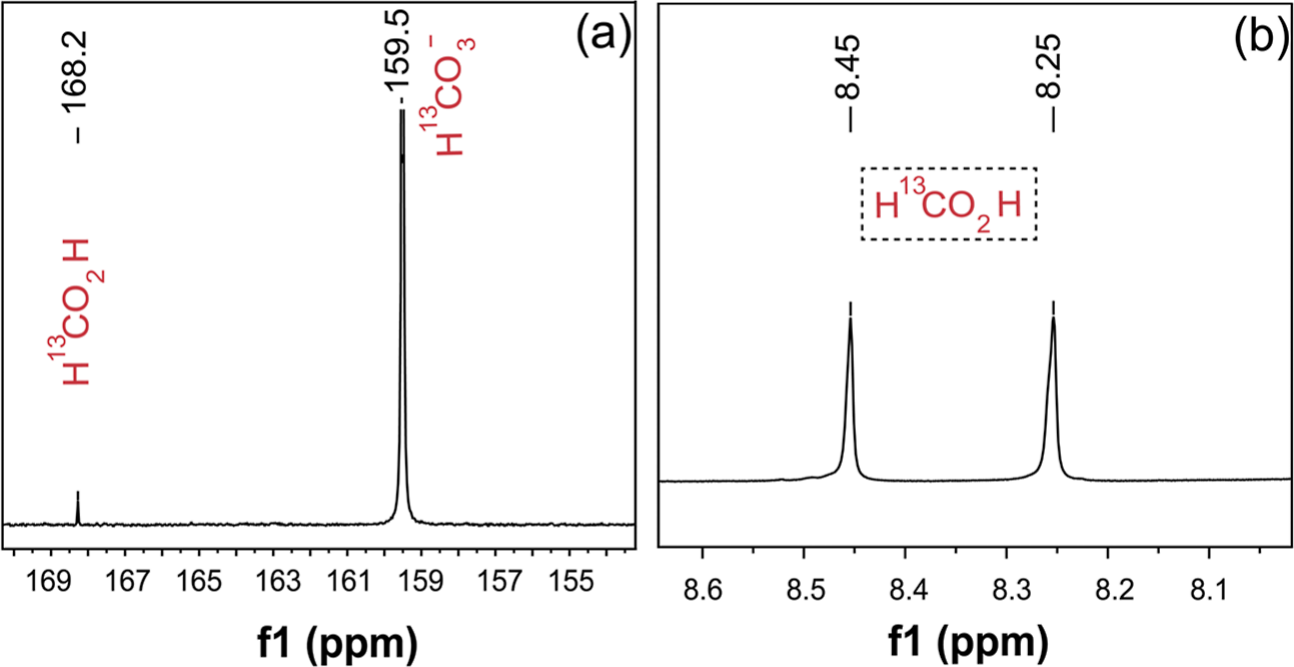
(a,b) ^13^C and ^1^H NMR spectra of the crude reaction mixture of Fe_2_O_3_ in BMMIm.MeIm IL; the reaction was performed for 5 h under 1-bar ^13^CO_2_ in a Fischer–Porter reactor under LED (10 W). The observed doublet is assigned to protons bound to the ^13^C atom in H^13^COOH. NMRs were collected at a spinning rate of 35 MHz.

Furthermore, the relationship between formic acid yield and reaction time was investigated, revealing a rapid initial formation rate (Figure 6c). Approximately 38.2 μmol of formic acid was produced within the first hour of reaction, which gradually increased to 42.0 μmol and 42.3 μmol after 2 and 3 h, respectively. A more pronounced increase was observed between 3 and 5 h, with the yield reaching 55.4 ± 4.0 μmol. These results indicate that the catalytic system promotes fast initial generation of formic acid, followed by a steady growth phase, ultimately leading to a significantly higher yield at extended reaction times.

Recycling experiments revealed a gradual decrease in catalytic activity with successive cycles (Figure 6e). Because ^1^H and ^13^C NMR spectra of the recovered IL show no detectable decomposition products (Figures S8–S11, Supporting Information), chemical degradation of the IL is unlikely to be the dominant deactivation pathway. Instead, the activity loss is more plausibly attributed to the interfacial restructuring effects. In aqueous media and under irradiation, water molecules can partially disrupt the direct imidazolium-Fe_2_O_3_ interactions that stabilize the ordered ion-pair layer at the surface, converting contact ion-pair or sandwich-type adsorption motifs into more solvated, water-shielded configurations.^58^ Such reorganization is expected to weaken interfacial electric fields and electronic coupling, thereby reducing the charge-transfer efficiency. Additional contributions from partial IL desorption/leaching, loss of ion-pair organization, and product inhibition or surface fouling by accumulated formate species may also play a role. Consistent with the preservation of the interfacial chemistry, postreaction XPS still evidences Fe–N interactions (Figure 3c), indicating that the IL remains associated with the surface, albeit with modified organization. These observations suggest that the decline in performance arises primarily from reversible interfacial and microenvironmental changes rather than irreversible catalyst or IL decomposition.

### 2.3 Electronic Structure and Interfacial Modulation

Diffuse reflectance UV–vis analysis shows only minor changes in the apparent bandgap upon IL incorporation (∼2.24–2.18 eV), indicating that the bulk electronic structure of Fe_2_O_3_ remains largely unaffected. In contrast, Mott–Schottky measurements reveal a reproducible shift in flat-band potential (from ∼ −0.62 to ∼ −0.37 V vs Ag/AgCl) together with an increase in donor density, consistent with modification of the interfacial electronic environment. Mössbauer spectroscopy confirms preservation of the hematite phase with Fe^3+^ as the dominant species and only minor reduced Fe contributions (<10%), ruling out significant bulk restructuring. Complementary XPS, FTIR, and DFT analyses indicate strong IL–surface interactions and an organized ion-pair arrangement at the interface, which modulates local charge distribution and stabilizes charge carriers. Collectively, these results demonstrate that ILs act as interfacial electronic regulators, tuning band-edge alignment, carrier density, and surface states while leaving the intrinsic band structure largely unchanged. This interfacial modulation, rather than bandgap alteration, underpins the enhanced photocatalytic performance. To further probe how these interfacial electronic and microenvironmental effects translate into catalytic pathways, in situ spectroscopic analyses were performed under reaction conditions.

### 2.4 Investigation of the Mechanism

The CO_2_ photoreduction using Fe_2_O_3_ in BMMIm.MeIm IL solution was monitored under reaction conditions by Fourier transform infrared (FTIR) spectroscopy. As shown in Figure 8a, new peaks gradually appeared upon photo-irradiation as the illumination time increased from 0 to 210 min. The peak at 1318 cm^–1^ is ascribed to CO_3_^2–^*. The intermediate COOH* (1592 cm^–1^) is also detected, which might be possibly caused by the favorable proton capture capability of CO_2_^•–^ radicals, while a peak at 2272 cm^–1^ is assigned to the adsorbed CO_2_. Electron paramagnetic resonance (EPR) spectroscopy was employed to investigate the electron-transfer process (Figure 8b). The EPR spectrum of Fe_2_O_3_ in BMMIm.MeIm IL solution exhibited a weak signal at g = 3365 in the dark, which can be attributed to the imidazolium-cation radical [BMMIm]^•+^; the solution was saturated with CO_2_.^59^ Upon light illumination, the intensity of this signal increased, indicating electron transfer from Fe_2_O_3_ to the BMMIm.MeIm IL (Figure 8b).Based on the results of FTIR, ^13^C NMR, and EPR spectroscopy, the possible mechanism over Fe_2_O_3_ in BMMIm.MeIm IL solution can be proposed (Figure 8c). The reaction proceeds through the formation of imidazolium-carboxylate species similar to those observed in the TiO_2_–IL photocatalyst,^30^ which undergo homolytic cleavage to generate the imidazolium radical cation [BMMIm] ^•+^ and CO_2_ radical anion ([CO_2_]^•‑^). On the other hand, the in situ generated reduced Fe species can reduce CO_2_ and generate the Fe^I^–CO_2_^•‑^ species.

**Figure 8:**
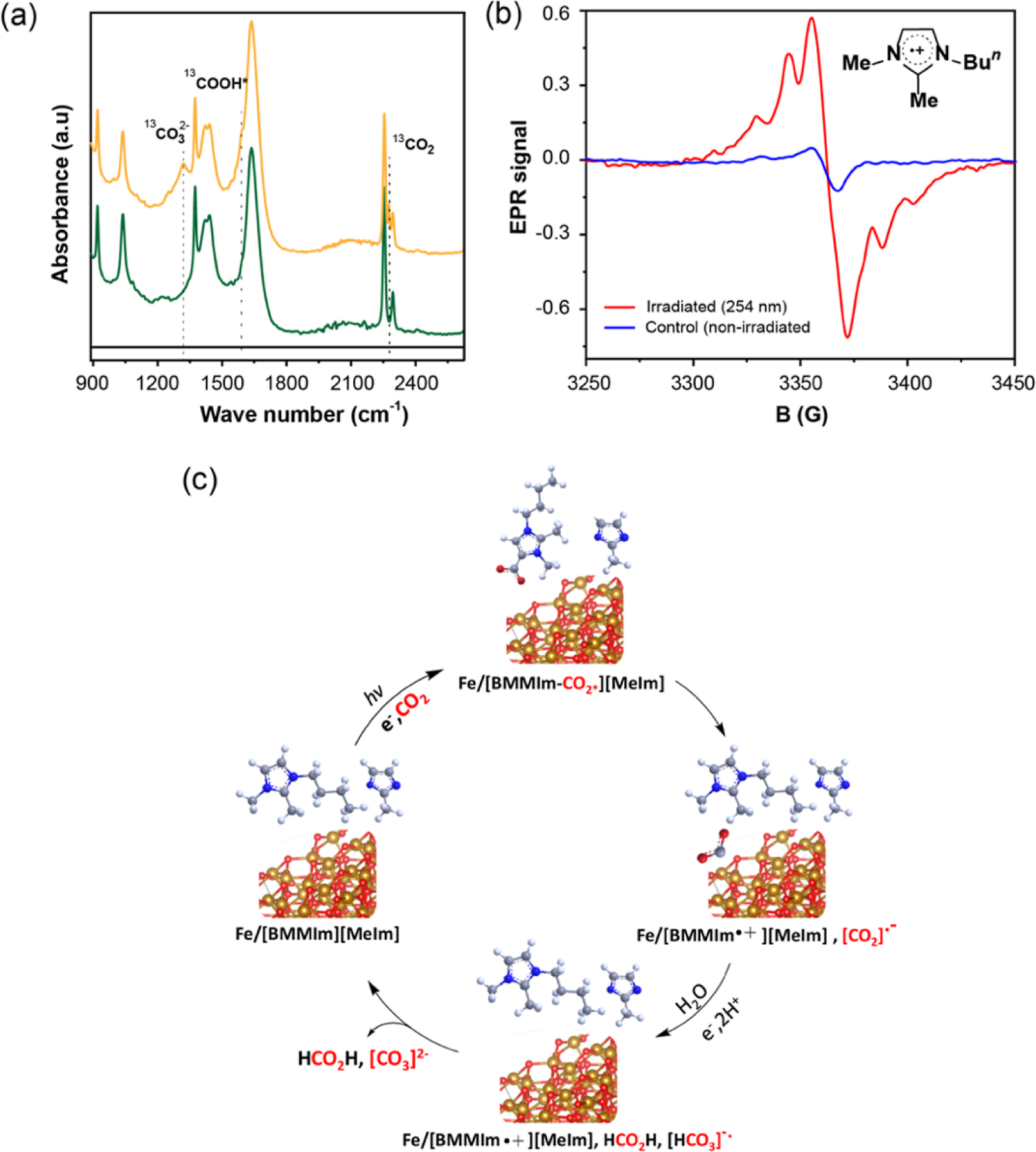
FTIR spectra of Fe_2_O_3_ in BMMIm.MeIm/CH_3_CN/H_2_O using ^13^CO_2_, (b) EPR analysis of Fe_2_O_3_ in BMMIm.MeIm/CH_3_CN/H_2_O saturated with CO_2_, and (c) proposed mechanism.

The formed CO_2_ radical anion captures protons to generate formic acid and bicarbonate species. The ^13^C NMR spectrum showed a peak at 159 ppm (Figure 7a), confirming the formation of bicarbonate (HCO_3_^–^).^60^ These bicarbonate species are subsequently consumed during the reaction, leading to the generation of neutral BMMIm.MeIm and carbonates (CO_3_^2–^).

The spectroscopic data provide evidence for light-induced interfacial charge transfer processes but do not uniquely define the elementary steps. EPR measurements reveal paramagnetic species assignable to imidazolium-derived radicals, FTIR identifies CO_2_-related intermediates, and XPS confirms strong interactions between the basic anion and the Fe_2_O_3_ surface. However, the presence of radical signatures alone does not unambiguously establish their direct catalytic participation. Consistent with this, no decomposition or coupling products of the IL are detected by ^1^H or ^13^C NMR after catalysis, indicating that the IL remains chemically intact (Figures S8–S11).

We therefore interpret the IL effect primarily within the well-established framework of interfacial electronic and microenvironmental modulation.^58,61,62^

ILs are known to modify semiconductor band energetics, enhance interfacial electric fields, stabilize charged intermediates, and regulate local proton activity, thereby facilitating selective multielectron CO_2_ reduction.^30^ In addition, ^13^C NMR and IR measurements provide direct evidence for CO_2_ capture via bicarbonate formation, demonstrating that the IL participates in CO_2_ adsorption and buffering of the reaction medium. Such buffering shifts the equilibrium toward two-electron products, including formate/formic acid, as previously reported for IL-assisted CO_2_ reduction systems.^63^ In this context, the IL acts as an interfacial regulator and chemical microenvironment rather than a sacrificial redox partner. Mechanisms involving transient imidazolium radicals, *N*-heterocyclic carbene species, or IL-CO_2_ adducts remain chemically plausible and consistent with the known reactivity of imidazolium systems but should be regarded as conditional pathways whose quantitative contributions cannot be conclusively established here. Accordingly, we describe these routes as possible contributors, rather than definitive mechanistic steps.

### 2.5 Prototype Outdoor Testing

To demonstrate operation under natural sunlight, the experiment was conducted at the Department of Chemistry, Universidade Federal de Goiás (UFG), on 18 March 2025 (Figure 9). Liquid aliquots were collected at regular time intervals for formic acid quantification, and the reaction temperature was monitored throughout the experiment (Figure 9c).

**Figure 9:**
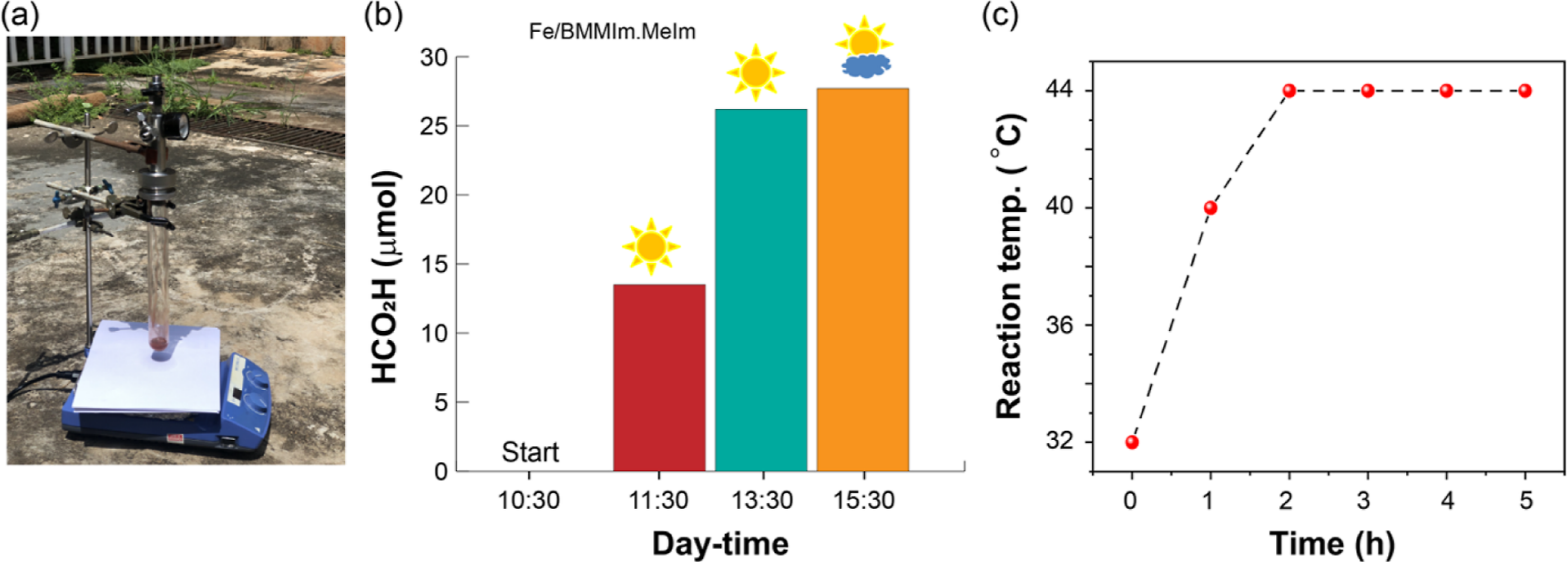
Natural daylight experiments: (a) photograph of the experimental setup; (b) formic acid generation as a function of time; and (c) monitoring of reaction medium temperature over the experimental period. Cat. (100 mg), IL (0.75 mmol), CO_2_ (1.0 bar, 200 mL reactor), CH_3_CN (2.5 mL), and H_2_O (0.5 mL).

The sunlight intensity remained relatively constant, except during the third hour, when a brief cloudy period occurred. After 1 h, 13.5 μmol (135 μmol.g^–1^) of formic acid was produced, indicating the rapid initiation of CO_2_ reduction. As the reaction proceeded, the concentration of formic acid increased steadily to 26.2 μmol (262 μmol.g^–1^) after 3 h. At 5 h, 27.7 μmol (277 μmol.g^–1^) of formic acid was obtained (Figure 9b). This slight increase in yield observed between 3 and 5 h is attributed to partial cloud cover during this interval. Passing or partially covering clouds primarily reduce the intensity of direct solar irradiation in the ultraviolet and visible regions, which are responsible for photocatalyst excitation, while the infrared component remains comparatively less affected. As a result, transient decreases in photon flux led to temporary reductions in charge-carrier generation and reaction rates. Although real-time spectral irradiance measurements were not available during these experiments, the consistent activity observed under fluctuating illumination conditions demonstrates the robustness of the IL/Fe_2_O_3_ system under practical sunlight exposure.

## 3 Conclusion

Iron oxide microrods combined with ILs constitute an efficient platform for the selective photoreduction of CO_2_ to formic acid in aqueous media without the use of any organic sacrificial agent. This subtle structural modification correlates with enhanced photoactivity. DFT calculations show that, on the Fe_2_O_3_ surface, the imidazolium cation of BMMIm.MeIm orients nearly parallel while the imidazolate anion adopts a perpendicular configuration, enabling efficient interfacial charge transfer. The ILs primarily modulate band-edge alignment and interfacial electronic structure, while only minor changes in the apparent optical bandgap are observed. Among the ILs tested, BMMIm.MeIm afforded the highest performance, yielding 55.4 μmol of formic acid with a rate of 110.8 μmol.g^–1^.h^–1^ with >99% selectivity under LED irradiation, while BMIm.OAc produced 16.1 μmol with the rate of 32.2 μmol.g^–1^.h^–1^ under identical conditions. Mechanistic evidence supports a pathway involving the formation of [CO_2_]•^–^ and imidazolium-cation radical species as reactive intermediates. Notably, the system also operates efficiently under natural sunlight, generating 27.7 μmol (277 μmol·g^–1^) of formic acid with excellent selectivity. Overall, IL-modified Fe_2_O_3_ microrods demonstrate how tailored interfacial ion–surface interactions can modulate electronic structure, enhance charge-transfer kinetics, and drive efficient solar-assisted CO_2_ reduction. These findings highlight the potential of IL-semiconductor hybrid systems as robust photocatalysts for sustainable C_1_ fuel production.

## 4 Experimental Section

All the ILs, 1,2-dimethyl-1-*n*-butyl-imidazolium 2-methylimidazolate (BMMIm.MeIm), 1-*n*-butyl-3-methylimidazolium acetate (BMIm.OAc, potassium 2-methylimidazolate (K.MeIm), tri-*n*-butyl-ethyl-phosphonium imidazolate (But)_3_EP.Im), and 1-*n*-butyl-3-methylimidazolium tetrafluoroborate (BMIm.BF_4_), were prepared, as reported previously.^64,65^ Freshly prepared ILs were used for catalytic tests. CO_2_ (>99.999%) was purchased from White-Martins Ltd., Brazil. NMR analyses were performed on a Bruker-III 500 MHz spectrometer. The reaction temperature was monitored at 1h intervals using an infrared thermometer (digital laser infrared thermometer). The lighting power density (LPD) of our cold white LED (10W) was about 18 mW/cm^2^. The size and diameter of the Pyrex tube of the Fischer–Porter reactor were 30 and 3.0 cm, respectively. NMR analyses were performed on a Bruker Ascend 400 MHz spectrometer. Using the zg pulse program (Bruker), the spectral window was 25 ppm centered at 4.7 ppm for all ^1^H NMR spectra. Sixteen scans were accumulated with a repetition time of 30 s and an acquisition time of 4.91 s (totaling 96k spectral points). Pulses were calibrated for each sample. The repetition time was estimated via an inversion–recovery experiment (pulse program t1ir1d). The FA yield was calculated from the molar ratio of FA generated relative to the amount of IL used. The apparent quantum efficiency (AQE) for formic acid production was evaluated using a 10 W LED (assuming a dominant emission wavelength of 450 nm), as reported earlier.^66^ In 1 h, the formic acid yield was determined to be 38.2 μmol by using 100 mg of catalyst.

### 4.1 Photocatalytic Tests

All photocatalytic experiments were performed in a 220 mL Pyrex glass Fischer–Porter reactor (Figure 9a). Typically, 0.1 g of catalyst was dispersed, followed by the addition of the desired amount of IL, CH_3_CN (2.5 mL), and deionized H_2_O (0.5 mL). The reactor was then purged with CO_2_ to remove air and subsequently filled with 1-bar CO_2_. The reaction medium was irradiated using either a 10 W cold white LED or a 365 nm LED. After 5 h of reaction, the gaseous products were analyzed by GC, and formic acid was quantified by NMR. The reaction mixture was centrifuged to remove the catalyst, and the supernatant (250–300 μL) was directly analyzed by ^1^H NMR using DMSO-*d*_6_ (400 μL) to determine the amount of formic acid produced.

## Supplementary Material



## Notes

Detailed information on the XRD, UV–vis, XPS, EPR analysis, NMR, and computational details of catalysts (PDF)
