# Supplementary material for: Iron Age: Ionic-Liquid-Mediated Interfacial Charge Transfer Enables Selective CO2 Photoreduction to Formic Acid on Iron Oxide
Source: J Am Chem Soc. 2026 May 17;148(21):22189–201. doi: 10.1021/jacs.6c04801 (PMC13244471; doi:10.1021/jacs.6c04801)
Supplement: Supplementary file 1 [file ja6c04801_si_001.pdf]

Supporting Information

Iron Age: Ionic-Liquid-Mediated Interfacial Charge Transfer Enables Selective CO_2 Photoreduction to Formic Acid on Iron Oxide

Muhammad I. Qadir,*[,§,†] Blendo A. da Silva,[†] Sherdil Khan,[‡] Renato B. Pontes,[¥] Fabiano S. Rodembusch,[§] Fabiano Mesquita,[‡] Brenno A. D. Neto,[¶,$] Paulo E. N. de Souza,[¶] Jairton Dupont*[,§,#]

§ Institute of Chemistry-Universidade Federal do Rio Grande do Sul-UFRGS-Av. Bento Gonçalves, 9500 Porto Alegre 91501-970, Porto Alegre, RS, Brazil.
† Instituto de Química-Universidade Federal de Goiás-UFG-Av. Esperança s/n, Câmpus Samambaia. 74690-900, Goiânia, Goiás, Brazil.
‡ Institute of Physic, Universidade Federal do Rio Grande do Sul (UFRGS), Av. Bento Gonçalves, 9500, Porto Alegre 91501-970, RS, Brazil.
¥ Instituto de Fisica-Universidade Federal de Goiás-UFG-Av. Esperança s/n, Campus Samambaia, 74690-900, Goiânia, Goiás, Brazil.
¶ Laboratory of Medicinal and Technological Chemistry, University of Brasília, Chemistry Institute (IQ-UnB), Campus Universitário Darcy Ribeiro, Brasília, Distrito Federal 70910-900, Brazil.
$ Universidade Estadual de Goiás, Molecular Sciences Graduate Programe, Anápolis, GO 75132-400, Brazil.
# Departamento de Bioquímica y Biología Molecular B e Inmunología Facultad de Química, Universidad de Murcia, P.O. Box 4021, E-30100 Murcia, Spain.

Contents **Pages**

1. XRD of iron oxide microrods 2
2. UV-Vis spectroscopic results of catalysts 2
3. XPS wide-scan spectra of catalysts 5
4. Mössbauer spectroscopy of catalysts 5
5. Computational Details 6
6. Photoreactors and temperature of reaction mixture 7
7. EPR analysis of catalysts 7
8. Literature comparison of catalysts 9
9. ^{1}H-NMR of crude reaction mixture containing BMMIm.MeIm IL/solvent/H_2O mixture after photoreduction of CO_2 10
10. ^{13}C-NMR of crude reaction mixture containing BMMIm.MeIm IL/solvent/H_2O after photoreduction of CO_2 11
11. ^{1}H-NMR of crude reaction mixture containing BMMIm.MeIm IL/solvent/H_2O after photoreduction in enriched $^{13}CO_2$ 12
12. ^{1}H and ^{13}C NMR of soley IL/solvent reaction mixture without Fe_2O_3 after irradiation 13
13. Light source characterization 14
14. UV-Vis spectrum of the ILs 15
15. BET analysis of catalysts 15
16. References 15

1. XRD of iron oxide microrods

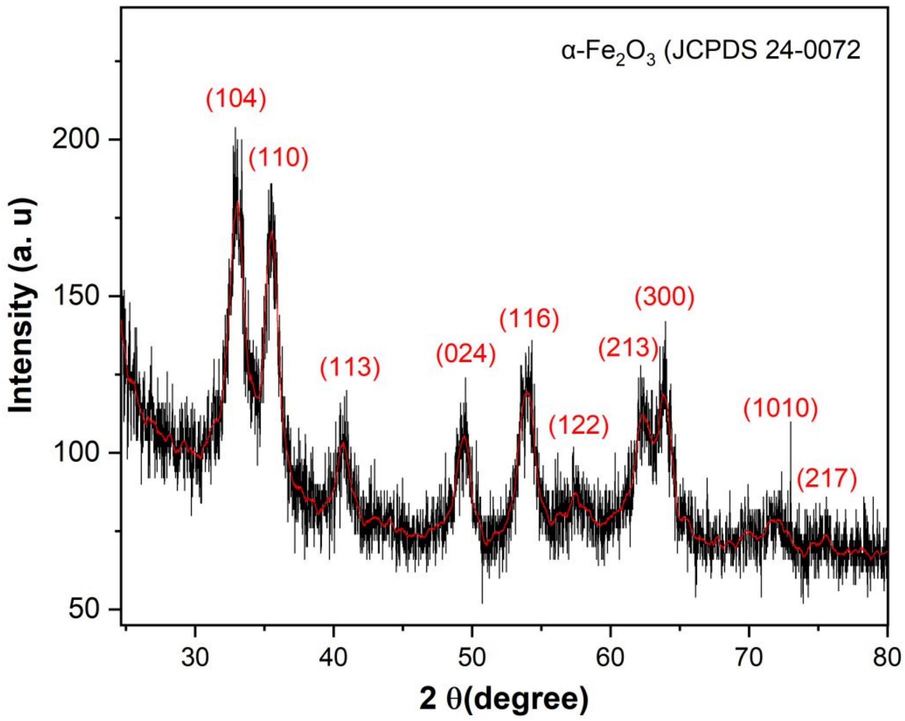

Figure S1. PXRD of pristine Fe_2O_3 microrods.

2. UV-Vis spectroscopic results of catalysts

Diffuse reflectance UV–Vis (DRUV) spectra were recorded using a Shimadzu UV-2450PC spectrophotometer equipped with an ISR-2200 integrating sphere. Measurements were carried out at room temperature over the 330-830 nm spectral range. Barium sulfate ($BaSO_4$, Wako Pure Chemical Industries, Ltd.) was employed as the reference material for baseline correction in the solid state. The samples were analyzed in powdered form. The bandgaps were estimated using the lambda onset from the solid-state absorbance spectra [1] and Tauc plots,[2] considering a direct electronic transition [3].Photoluminescence (PL) spectra were obtained using a Shimadzu RF-5301 spectrofluorometer fitted with a solid-state sample holder positioned at an angle to minimize interference from reflected excitation light reaching the emission monochromator. Photoluminescence measurements were performed at various excitation wavelengths (240-540 nm, 20 nm step) to investigate the photophysical properties of the iron oxides.

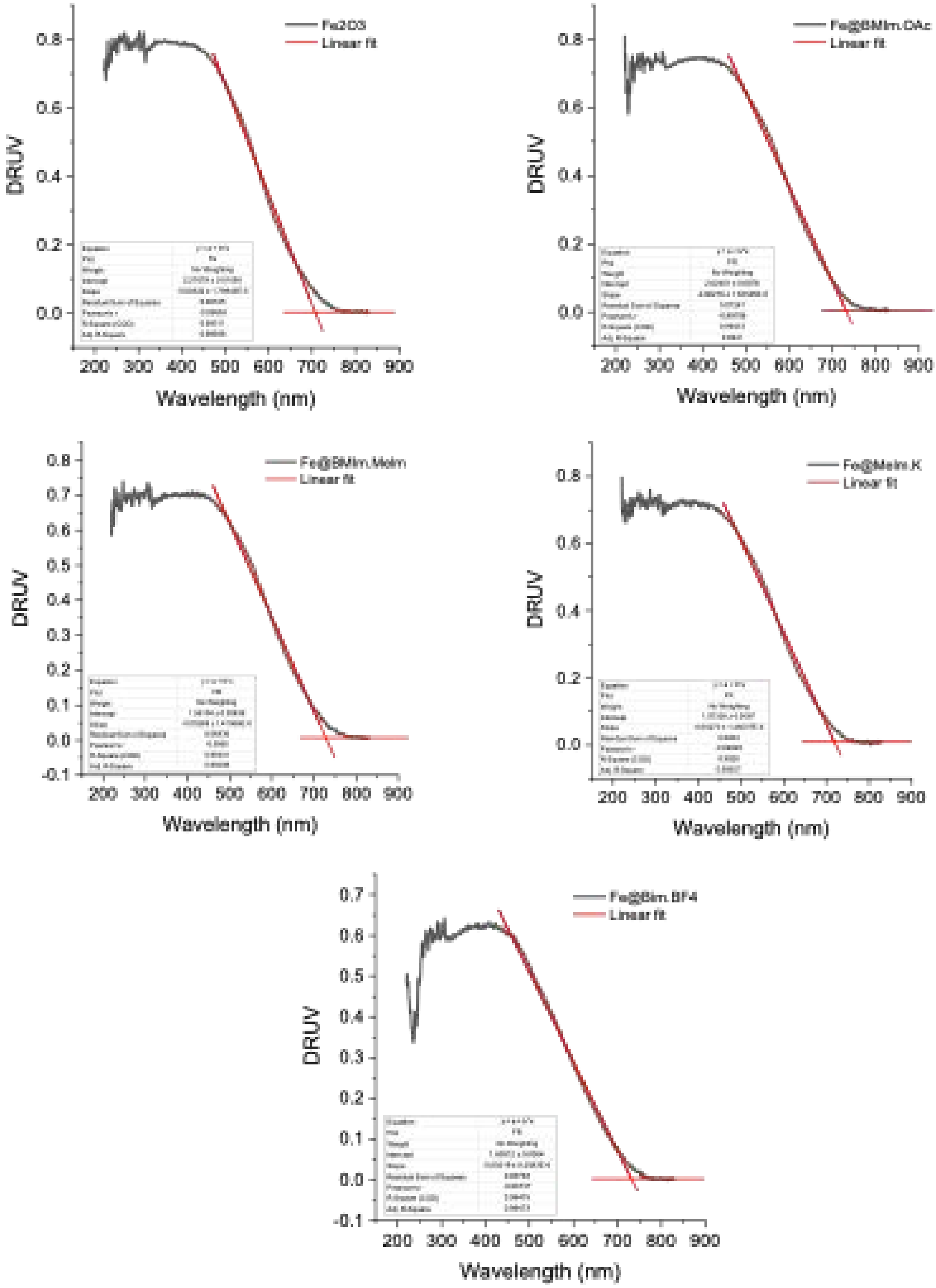

Figure S2. DRUV spectra (330-830 nm) of the Fe_2O_3, Fe@BMIm.OAc, Fe@BMMIm.MeIm, Fe@MeIm.K and Fe@BMIm.BF_4.

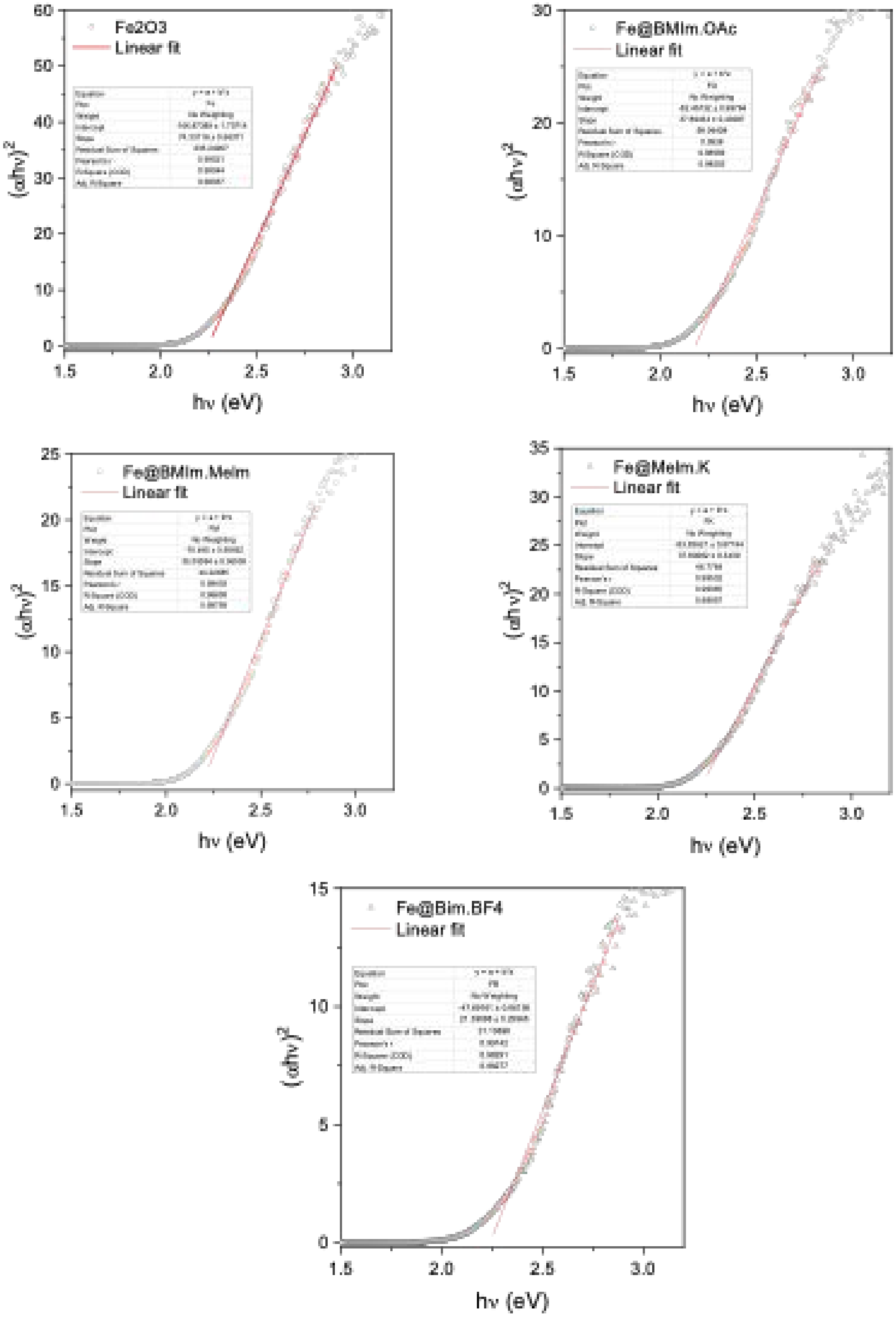

Figure S3. Tauc plot of the Fe_2O_3, Fe@BMIm.OAc, Fe@BMMIm.MeIm, Fe@MeIm.K and Fe@BMIm.BF_4 samples and respective linear fit used for band gap estimation.

3. XPS wide-scan spectra of catalysts

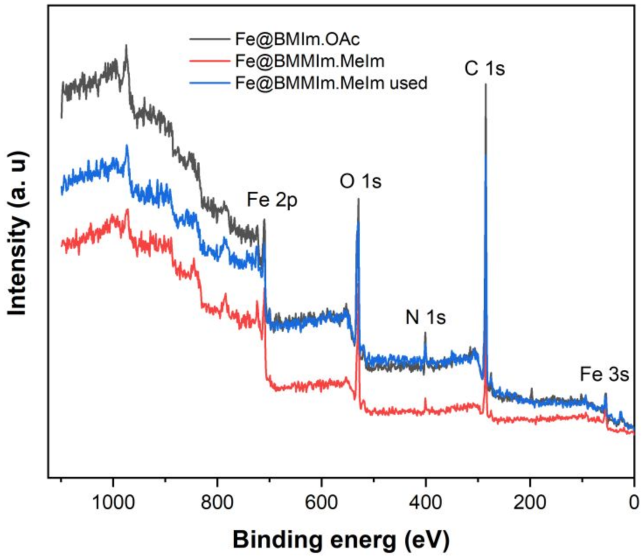

Figure S4. XPS wide-scan spectra of catalysts.

4. Mössbauer spectroscopy of catalysts

The Mssbauer spectra were recorded using a spectrometer operating in constant acceleration transmission mode with a nominal 50 mCi^{57}Co(Rh) source. Approximately 10 mg of each sample was used to optimize signal intensity.

Table S1. Summary of the results from the analysis of the Mössbauer spectra. The site num- bering refers to the hyperfine parameters found in the samples, where Site 1 and Site 2 are related to the superparamagnetic responses of hematite and metallic iron (minority phase), respectively.

	**Fe_2O_3**	**Fe@BMIm.OAc**	**Fe@BMMMIm.MeIm**	**Fe@K.MeIm**
H_{Eff1} (M)	-	-	-	-
EQ_1	0.65(0.02)	0.58(0.02)	0.61(0.02)	0.62(0.02)
IS_1	0.35(0.02)	0.35(0.02)	0.36(0.02)	0.35(0.02)
Larg.Lin. 1	0.52(0.01)	0.53(0.01)	0.56(0.01)	0.51(0.01)
Area 1	1	0.9051	0.9252	0.9404
H_{Eff2} (M)	-	-	-	-
EQ_2	-	0.23(0.02)	0.30(0.02)	0.34(0.02)
IS_2	-	0.00(0.02)	0.00(0.02)	0.00(0.02)
Larg.Lin. 2	-	0.56(0.01)	0.38(0.01)	0.31(0.01)
Area 2	-	0.0949	0.0748	0.0546
X^2				

5. Computational Details

All the spin-polarized DFT calculations were carried out using the widely used plane wave Vienna ab initio simulation package, which treats the interactions between ions and electrons by the projector augmented wave method (PAW) [4-7]. The generalized gradient approxima-tion (GGA) with the Perdew–Burke–Ernzerhof (PBE) exchange-correlation functional was used in this study with a kinetic cut-off energy of 500 eV [8]. The Brillouin zone was sampled by using a Monkhorst-Pack scheme with a grid size of 4 × 4 × 1 [9]. To treat the on-site Coulomb and exchange interaction of the strongly localized 3d electrons of metal atoms, here we employed the DFT + U in the Dudarev's formalism [10]. The DFT + U is a widely used method to describe transition metal-related studies. We consider an U_{eff} of 4.0 eV for Fe atoms, that were found appropriate in previous investigations [11]. The unit cell of bulk Fe_2O_3 (a = b= 5.05 Å and c = 13.76 Å) was used to construct the Fe_2O_3 (110) surface. In this investigation, Fe_2O_3 (110) was modeled from the optimized bulk, consisting of nine atomic layers in which the bottom three atomic layers were fixed during the structural optimization. A vacuum layer of 15 Å in the *z*-direction was included in the surface model to prevent the interactions between the repeated slabs. The geometry relaxation was stopped when the electronic energy tolerance (10^{-6} eV) was reached, and the residual forces on each atom were less than 0.01eV/Å. The van der Waals interactions (vdW) are included by using the semi-empirical D3 method [12].

6. Photoreactors and temperature of reaction mixture

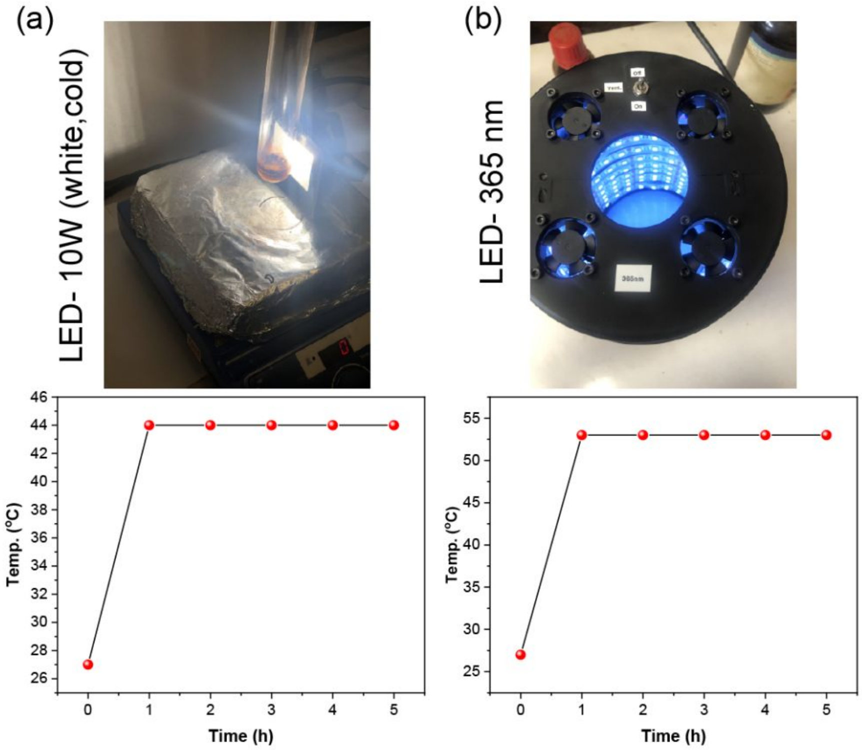

Figure S5. Photo-lamps and the temperature graph during reactions. The 365 nm UV LED (Model M5050N1UVS16G12-365) was purchased from Shenzhen Silverlight Technologies Co., Ltd. (China). The power supply used for photo-lamps was 220 V. The size and diameter of the Pyrex tube for reactor were 30 and 3.0 cm

7. EPR analysis of catalysts

All EPR measurements were performed using a Bruker EMXplus spectrometer (Germany) equipped with an X-band (9 GHz) high-sensitivity cavity (Bruker ER 4119HS, Germany). Frozen samples were placed inside a quartz-finger Dewar (Noxygen, Germany) filled with liquid nitrogen. A 400 µL aliquot of the aqueous ionic liquid solution was collected, transferred to a 1 mL de-capped syringe, and frozen in liquid nitrogen. The frozen cylindrical samples were then positioned inside the resonator for measurement. EPR spectra were recorded at -196 °C, ensuring identical sample volumes for all measurements. Irradiated samples were exposed to 254 nm light before analysis at -196 °C. The instrumental settings were as follows: 2 mW microwave power, 10 G

modulation amplitude, 100 kHz modulation frequency, 1000 G sweep width, 3365 G central field, and 50 s sweep time.

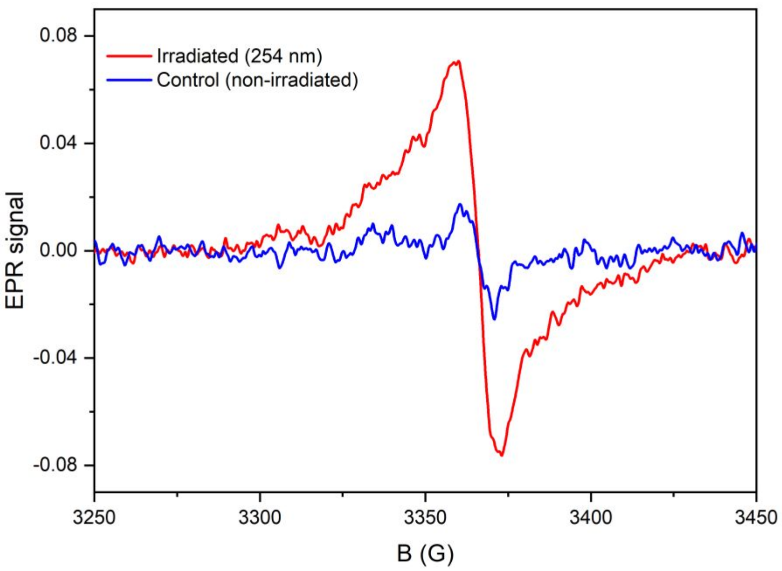

Figure S6. EPR spectra of before (blue) and after (red) irradiation (254 nm) in aqueous solution of iron oxide in BMIm.OAc/CH_3CH/H_2O upon CO_2 reaction.

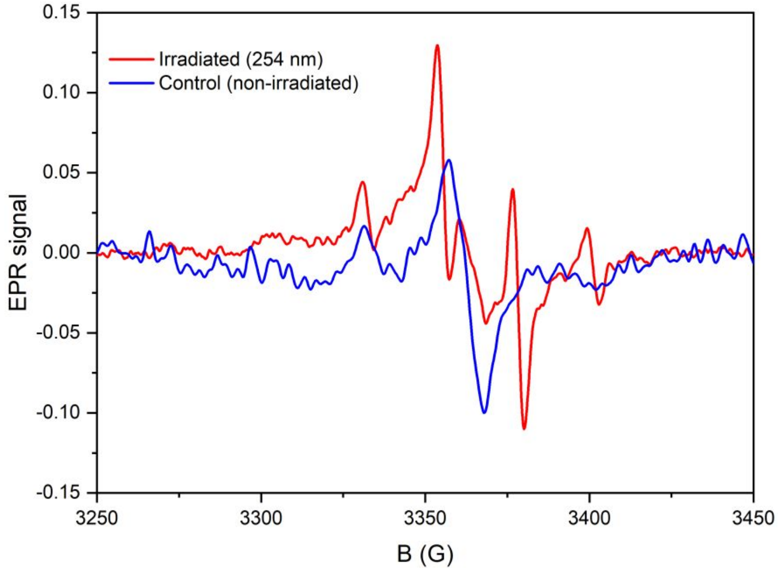

Figure S7. EPR spectra of before (blue) and after (red) irradiation (254 nm) in aqueous solution of iron oxide in K.MeIm/CH_3CH/H_2O upon CO_2 reaction.

8. Literature comparison of catalysts

Table S2: Heterogeneous photocatalysts for CO_2 photoreduction to formic acid/formats

S.No	Cat	Solvent	Lamp	Yield μmol	AQY (%)	Ref.
1	**RuRu'/**Ag/C 3 N4	DMA/TEOA	n.p	42.3	5.2	[13]
2	**RuP**/Ag/NS-C_3N_4	DMA and TEOA	400W	48.0	4.2	[14]
3	FeOOH/Al2O3/**Ru**^{II}	DMA/BNAH	400W	10.0	n.p	[15]
4	Eu-bpy-**Ru**-40%-CuCl2	CH_3CN/H_2O/TEA	300W	6.2	n.p	[16]
5	MOF-253–**Ru**$(CO)_2Cl_2$/ **Ru**^{II}	MeCN/TEOA	300W	8.23	n.p	[17]
6	AUBM-4/ **Ru(**cptpy$)_2$	MeCN/TEOA	150W	44.0	n.p	[18]
7	Eu-**Ru**(phen)3-MOF	MeCN/TEOA	300W	47.0	n.p	[19]
8	Fe-Zn-MOFs	H_2O	300W	12.0	0.035	[20]
9	zirconium–porphyrin MOF	MeCN/TEOA	300W	30.0	n.p	[21]
10	Cu/$BiYO_3$	NAOH/Na_2SO_3	300W	2.0	n.p	[22]
**11**	**Fe_2O_3**	**BMMIm.MIm/ MeCN/H_2O**	**LED**	**55.4 ± 4.0**	**4.4**	**our**

*n.p= Not provided

9. ^{1}H-NMR of crude reaction mixture containing BMMIm.MeIm IL/solvent/H_2O mixture after photoreduction of CO_2

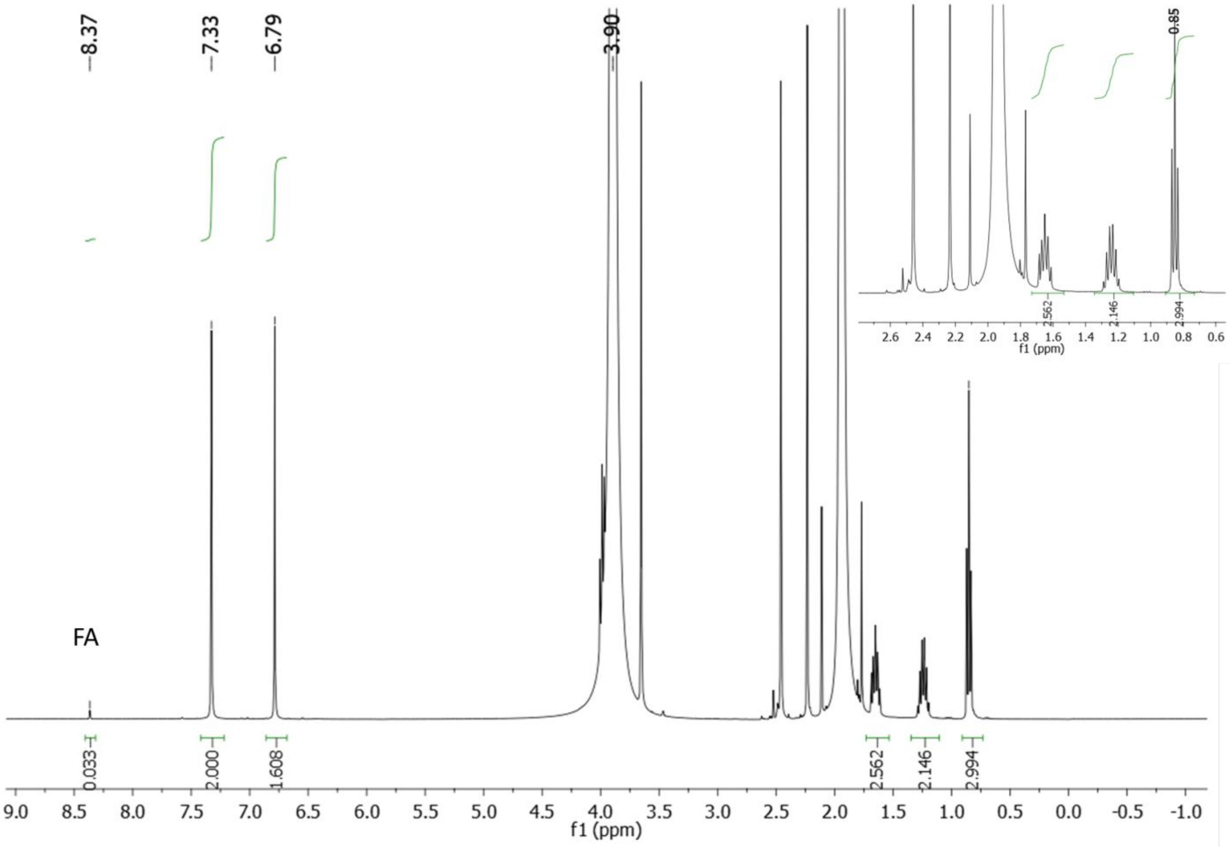

Figure S8. NMR spectra of crude reaction mixture of iron micro-rods in BMMIm.MeIm/CH_3CN/H_2O after photoreaction. FA: formic acid. Cat. (100 mg), IL (0.75 mmol), CO_2 (1.0 bar, 200 mL reactor) CH_3CN (2.5 mL), H_2O (0.5 mL), temp. (27±2 °C) and time (5 h). Catalyst was removed by centrifugation before NMR analysis. NMRs were collected at spinning rate of 35 MHz. ^{1}H-NMR shows that there is no degradation of BMMIm.MeIm IL during photo-reduction, as can been seen in zoom part. LED 10 W

10. ^{13}C-NMR of crude reaction mixture containing BMMIm.MeIm IL/solvent/H_2O after photoreduction of CO_2

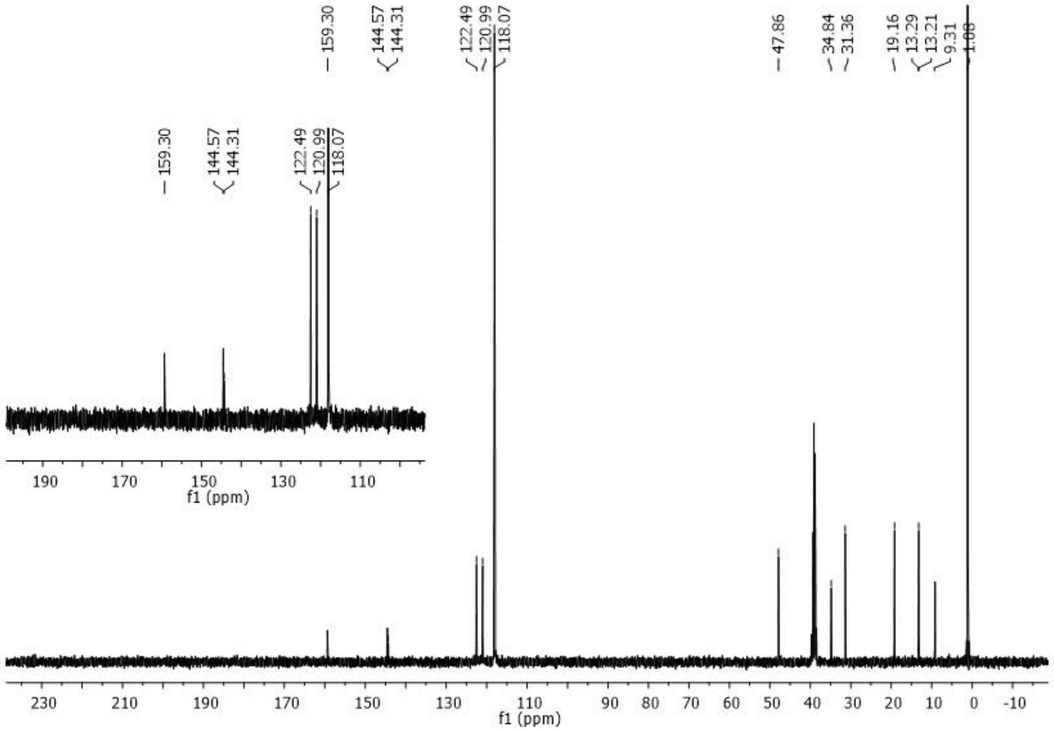

Figure S9. ^{13}C NMR spectra of crude reaction mixture of iron micro-rods in BMMIm.MeIm/CH_3CN/H_2O after photoreaction. FA: formic acid. Cat. (100 mg), IL (0.75 mmol), CO_2 (1.0 bar, 200 mL reactor) CH_3CN (2.5 mL), H_2O (0.5 mL), temp. (27±2 °C) and time (5 h). Catalyst was removed by centrifugation before NMR analysis. NMRs were collected at spinning rate of 35 MHz. ^{13}C-NMR shows that there is no degradation of BMMIm.MeIm IL during photo-reduction, as can been seen in zoom part. LED 10W

11. ^{1}H-NMR of crude reaction mixture containing BMMIm.MeIm IL/solvent/H_2O after photoreduction in enriched $^{13}CO_2$

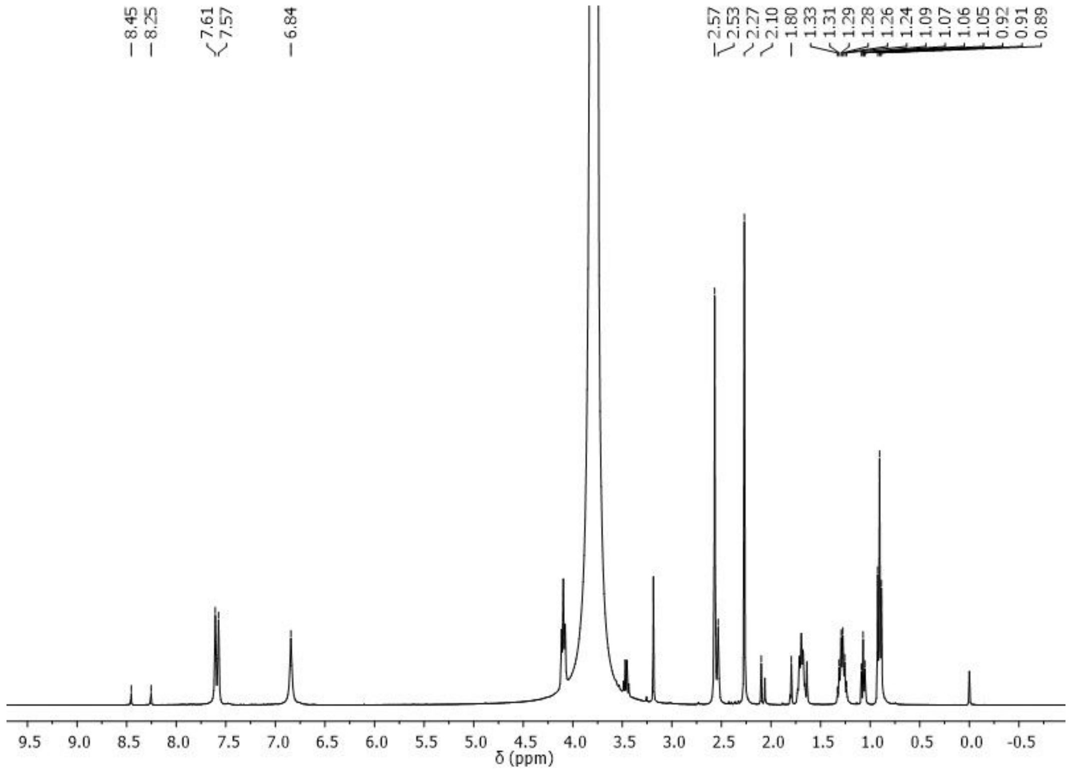

Figure S10. ^{1}H NMR spectra of crude reaction mixture of iron micro-rods in BMMIm.MeIm/CH_3CN/H_2O after photoreaction. FA: formic acid. Cat. (100 mg), IL (0.75 mmol), CO_2 (1.0 bar, 200 mL reactor) CH_3CN (2.5 mL), H_2O (0.5 mL), temp. (27±2 °C) and time (5 h). Catalyst was removed by centrifugation before NMR analysis. NMRs were collected at spinning rate of 35 MHz. LED 10W.

12. ^1H and ^{13}C NMR of soley IL/solvent mixture without Fe_2O_3 after irradiations

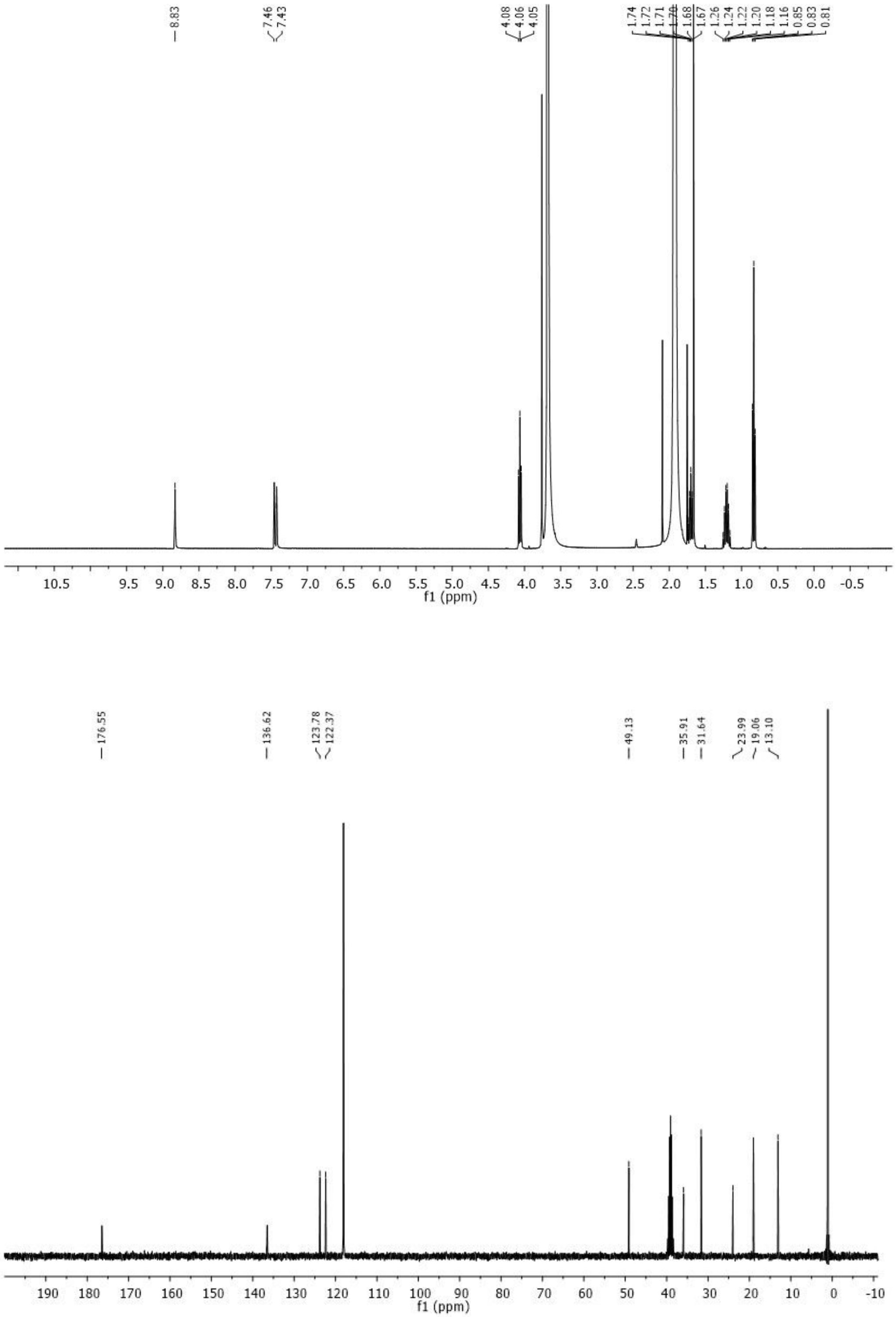

Figure S11. ^1H and ^{13}C NMR spectra of reaction mixture of BMIm.OAc/CH_3CN/H_2O after photoreaction. IL (0.75 mmol), CO_2 (1.0 bar, 200 mL reactor) CH_3CN (2.5 mL), H_2O (0.5 mL), temp. (27±2 °C) and time (5 h). DMSO-d6 was NMR solvent. NMRs were collected at spinning rate of 35 MHz. LED- 365 nm

13. Light source characterization

Emission spectrum of the 10 W LED lamp was obtained using an Ocean Optics FLAME-S-XR1 spectrophotometer, previously calibrated with a DH-3 Plus Enhanced Calibration UV-Vis-NIR light source connected via a QP600-1-UV-Vis optical fiber. The light source was positioned 5.0 cm from the source.

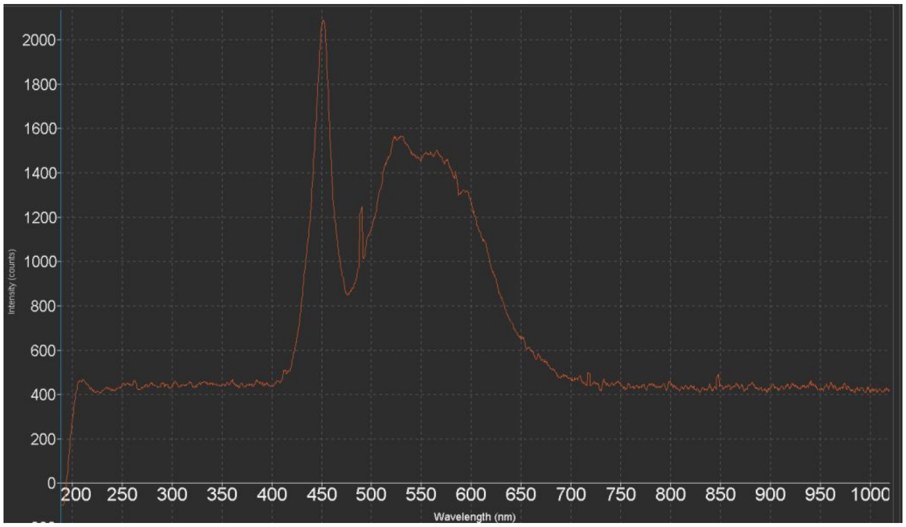

Figure S12. Emission spectrum of the LED 10W lamp. The spectrum was recorded under operating conditions, showing a maximum centered at 450 nm with a narrow bandwidth.

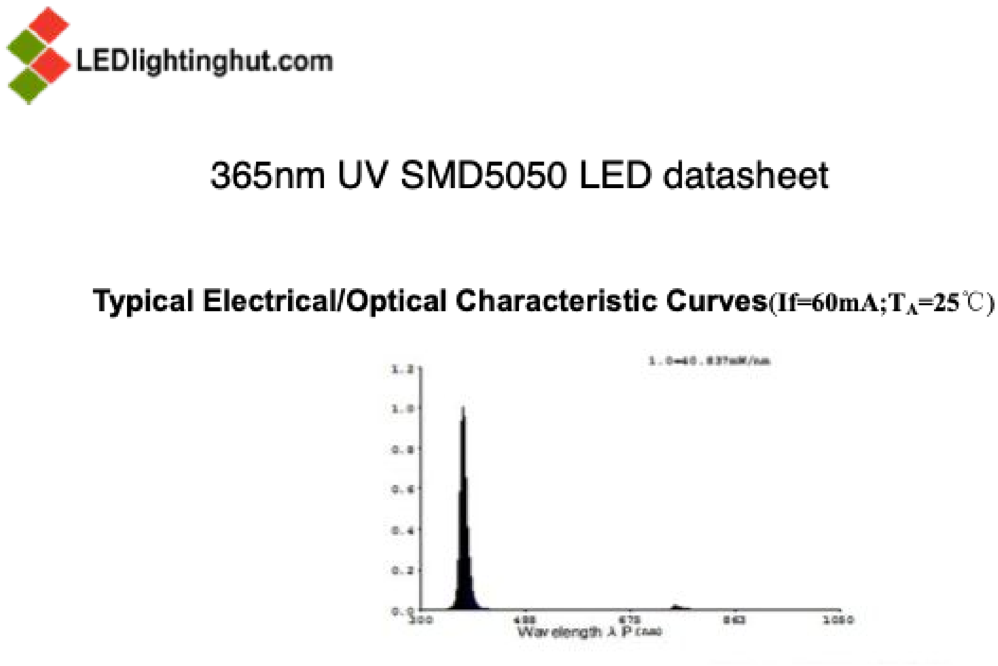

Figure S13. Specifications and emission spectrum of the 365 nm LED.

14. UV-Vis spectrum of the ILs

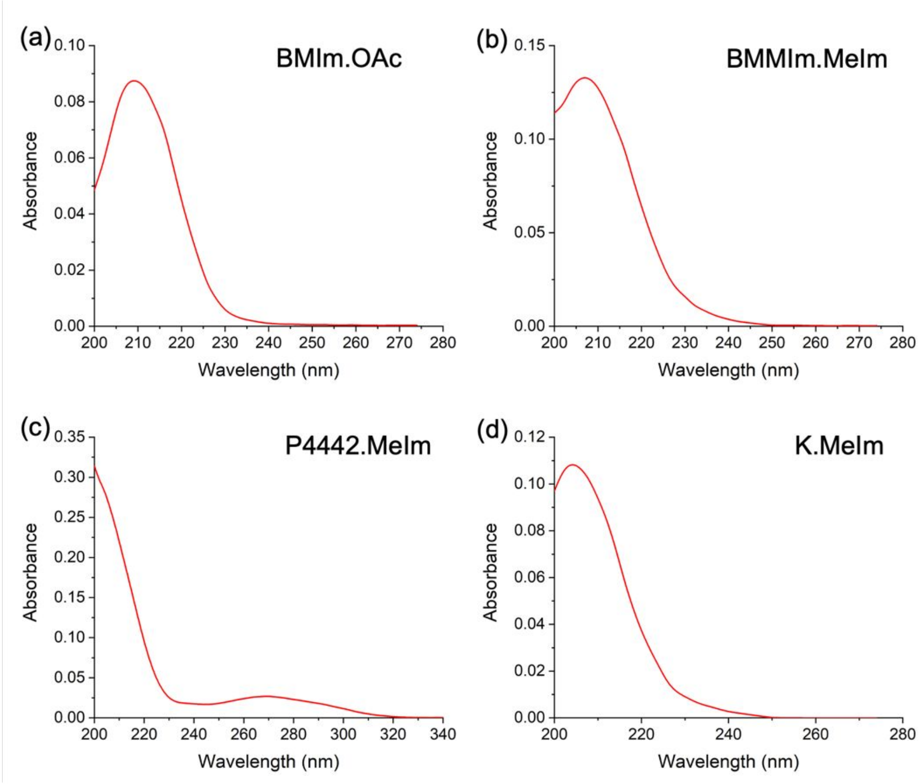

Figure S14. UV-Vis spectrum of the ILs

15. BET analysis of catalysts

Table S2. Physiochemical properties of catalysts

Entry	Cat.	Surface area (m^2/g)[a]	Pore size (nm)[b]	Pore volume (m^3/g)[b]
1	Fe_2O_3	137.1	4.2	0.14
2	Fe@BMMIm.MeIm	16.8	12.2	0.05
3	Fe@BMMIm.OAc	3.1	3.5	0.002

[a] Calculated from N_2 sorption using BET method. [b] Calculated from the N_2 adsorption and sorption isotherm using BJH method.

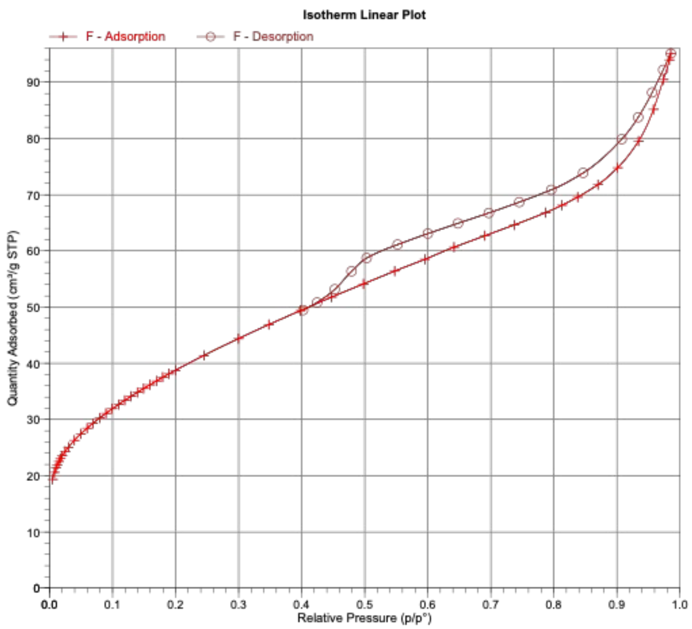

Figure S15. Nitrogen adsorption/desorption isotherms of the Fe_2O_3 catalyst

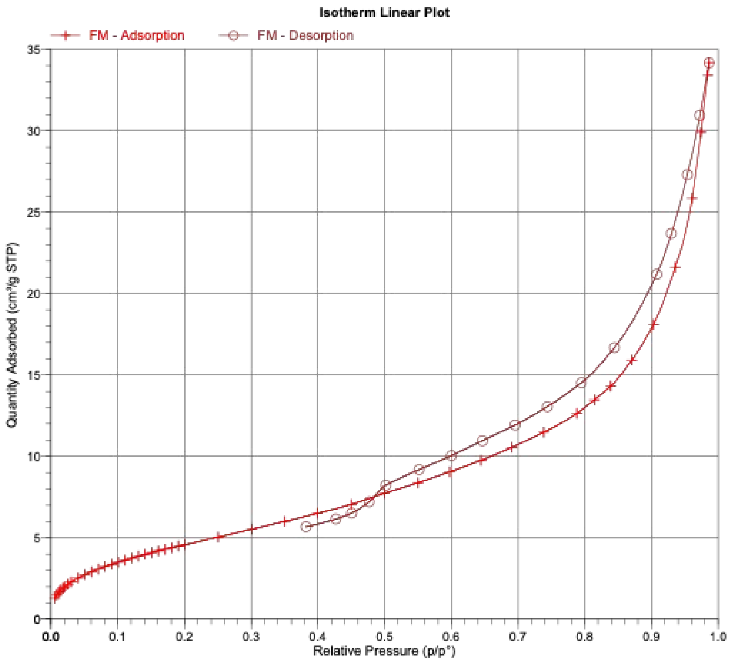

Figure S16. Nitrogen adsorption/desorption isotherms of the Fe@BMMIm.MeIm catalyst

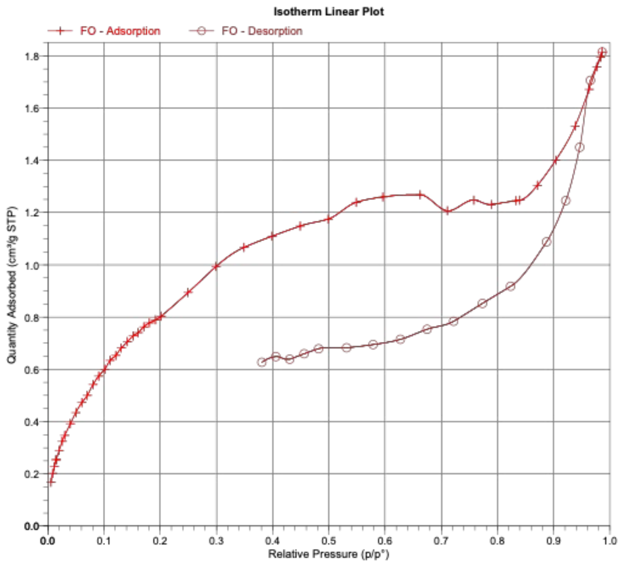

Figure S17. Nitrogen adsorption/desorption isotherms of the Fe@BMIm.OAc catalyst

16. References

1. A. M. Wallace, C. Curiac, J. H. Delcamp, R. C. Fortenberry, "Accurate determination of the onset wavelength (λonset) in optical spectroscopy," *J. Quant. Spectrosc. Radiat. Transfer* 265, no. (2021): 107544
2. J. Tauc, "Optical properties and electronic structure of amorphous Ge and Si," *Mater. Res. Bull.* 3, no. 1 (1968): 37-46
3. M. Alagiri, S. B. A. Hamid, "Green synthesis of α-Fe2O3 nanoparticles for photocatalytic application," *Journal of Materials Science: Materials in Electronics* 25, no. 8 (2014): 3572-3577
4. G. Kresse, J. Hafner, "Ab initio molecular-dynamics simulation of the liquid-metal--amorphous-semiconductor transition in germanium," *Physical Review B* 49, no. 20 (1994): 14251-14269
5. G. Kresse, J. Furthmüller, "Efficiency of ab-initio total energy calculations for metals and semiconductors using a plane-wave basis set," *Computational Materials Science* 6, no. 1 (1996): 15-50
6. G. Kresse, J. Hafner, "Ab initio molecular dynamics for liquid metals," *Physical Review B* 47, no. 1 (1993): 558-561
7. G. Kresse, D. Joubert, "From ultrasoft pseudopotentials to the projector augmented-wave method," *Physical Review B* 59, no. 3 (1999): 1758-1775
8. A. D. Becke, "Density-functional exchange-energy approximation with correct asymptotic behavior," *Physical Review A* 38, no. 6 (1988): 3098-3100
9. J. P. Perdew, K. Burke, M. Ernzerhof, "Generalized Gradient Approximation Made Simple," *Phys. Rev. Lett.* 77, no. 18 (1996): 3865-3868
10. S. L. Dudarev, G. A. Botton, S. Y. Savrasov, C. J. Humphreys, A. P. Sutton, "Electron-energy-loss spectra and the structural stability of nickel oxide: An LSDA+U study," *Physical Review B* 57, no. 3 (1998): 1505-1509

11. M. Gómez Toledo, S. Lopez Paz, S. Garcia Martin, E. Arroyo-de Dompablo, "Computational Investigation of the Potential SOFC Air Electrode YSr2Cu2FeO8-Δ," *ECS Meeting Abstracts* MA2023-01, no. 54 (2023): 78
12. M. E. Arroyo y de Dompablo, Y.-L. Lee, D. Morgan, "First Principles Investigation of Oxygen Vacancies in Columbite MNb2O6 (M = Mn, Fe, Co, Ni, Cu)," *Chem. Mater.* 22, no. 3 (2010): 906-913
13. R. Kuriki, H. Matsunaga, T. Nakashima, et al., "Nature-Inspired, Highly Durable CO2 Reduction System Consisting of a Binuclear Ruthenium(II) Complex and an Organic Semiconductor Using Visible Light," *J. Am. Chem. Soc.* 138, no. 15 (2016): 5159-5170
14. K. Maeda, D. An, C. S. Kumara Ranasinghe, et al., "Visible-light CO2 reduction over a ruthenium(ii)-complex/C3N4 hybrid photocatalyst: the promotional effect of silver species," *Journal of Materials Chemistry A* 6, no. 20 (2018): 9708-9715
15. D. An, S. Nishioka, S. Yasuda, et al., "Alumina-Supported Alpha-Iron(III) Oxyhydroxide as a Recyclable Solid Catalyst for CO2 Photoreduction under Visible Light," *Angew. Chem. Int. Ed.* 61, no. 26 (2022): e202204948
16. T.-C. Zhuo, Y. Song, G.-L. Zhuang, et al., "H-Bond-Mediated Selectivity Control of Formate versus CO during CO2 Photoreduction with Two Cooperative Cu/X Sites," *J. Am. Chem. Soc.* 143, no. 16 (2021): 6114-6122
17. D. Sun, Y. Gao, J. Fu, X. Zeng, Z. Chen, Z. Li, "Construction of a supported Ru complex on bifunctional MOF-253 for photocatalytic CO2 reduction under visible light," *Chem. Commun.* 51, no. 13 (2015): 2645-2648
18. M. Elcheikh Mahmoud, H. Audi, A. Assoud, T. H. Ghaddar, M. Hmadeh, "Metal–Organic Framework Photocatalyst Incorporating Bis(4′-(4-carboxyphenyl)-terpyridine)ruthenium(II) for Visible-Light-Driven Carbon Dioxide Reduction," *J. Am. Chem. Soc.* 141, no. 17 (2019): 7115-7121
19. Z.-H. Yan, M.-H. Du, J. Liu, et al., "Photo-generated dinuclear {Eu(II)}2 active sites for selective CO2 reduction in a photosensitizing metal-organic framework," *Nat. Commun* 9, no. 1 (2018): 3353
20. L.-Z. Dong, L. Zhang, J. Liu, et al., "Stable Heterometallic Cluster-Based Organic Framework Catalysts for Artificial Photosynthesis," *Angew. Chem. Int. Ed.* 59, no. 7 (2020): 2659-2663
21. H.-Q. Xu, J. Hu, D. Wang, et al., "Visible-Light Photoreduction of CO2 in a Metal–Organic Framework: Boosting Electron–Hole Separation via Electron Trap States," *J. Am. Chem. Soc.* 137, no. 42 (2015): 13440-13443
22. T. Su, H. Tian, Z. Qin, H. Ji, "Preparation and characterization of Cu modified BiYO3 for carbon dioxide reduction to formic acid," *Appl. Catal. B: Environ.* 202, no. (2017): 364-373
